# Simultaneous Inference of Flora‐Wide Nuclear and Plastid Phylogenies Using Angiosperms353 Sequence Capture Data

**DOI:** 10.1111/1755-0998.70202

**Published:** 2026-09-28

**Authors:** Sébastien Miche, Charles Pouchon, Camille Christe, Anna Marcionetti, Maya Wells, Yamama Naciri, Nicolas Wyler, Mathieu Perret

**Affiliations:** ^1^ Department of Plant Sciences University of Geneva Geneva Switzerland; ^2^ Jardin Botanique de Genève Chambésy Switzerland; ^3^ Department of Computational Biology University of Lausanne Lausanne Switzerland

**Keywords:** Angiosperms353 probe set, divergence time estimation, genomics, plant systematics, regional flora, species‐level phylogenetics, target sequence capture, tree of life

## Abstract

Accurate knowledge of phylogenetic relationships and divergence time among plant species composing a regional flora is crucial for understanding the origin of the biodiversity patterns we observe today. Despite advances in phylogenomics, resources available for reconstructing regional phylogenies are still largely incomplete and poorly resolved at the species level. We developed a workflow to assemble a phylogenetic dataset for a regional flora using Angiosperms353 target‐enrichment and high‐throughput genome sequencing. We sampled tissues from freshly collected plants and herbarium specimens representing the angiosperm diversity of the Geneva canton in Switzerland (1098 species). We used ORTHOSKIM to recover targeted nuclear genes and plastid loci from off‐target reads, estimated relationships and time divergence using IQTREE, weighted‐ASTRAL and treePL, and compared the results obtained from nuclear and plastid genes. We recovered a mean of 289 nuclear and 54 plastid genes for 1316 taxa, representing 92% of the species native to the Geneva canton. We demonstrate that nuclear genes provide increased resolution and phylogenetic support, but similar divergence time estimates compared to plastomes. Our workflow based on Angiosperms353 data provides a phylogenetic framework for an entire regional flora that is consistent with current systematic knowledge and more resolved and complete at the infrageneric level than trees obtained from phylogenetic resources available for angiosperms. This type of genomic data opens perspectives for fine‐scale biodiversity and conservation research that integrates evolutionary history.

## 1 Introduction

Large dated phylogenetic trees that encompass most of the lineages occurring in a given region or ecosystem have become essential resources for investigating how and when biotas evolved and assembled (Sauquet et al. 2009; Simon et al. 2009; Merckx et al. 2015; Dexter et al. 2017; Xing and Ree 2017; Antonelli et al. 2018; Pérez‐Escobar et al. 2022; Smyčka et al. 2022; Cano et al. 2022; Wootton et al. 2025). An access to regional phylogenetic datasets is also crucial for estimating the phylogenetic structures of species assemblages (Webb 2000; Cavender‐Bares et al. 2009) and the turnover of evolutionary diversity following land‐use intensification or climate change (Knapp et al. 2008; Thuiller et al. 2011; Spalink et al. 2018; Saladin et al. 2020; Franke et al. 2020). Additionally, phylogenies help defining meaningful units for conservation management and identifying species or regions that should be prioritized for conservation to prevent the disproportionate loss of evolutionary history (Forest et al. 2007; Mishler et al. 2014; Thornhill et al. 2016, 2017; Allen et al. 2019; Pipins et al. 2024). Therefore, our ability to construct robust and comprehensive regional phylogenies is pivotal in shaping our understanding of biodiversity and guiding conservation efforts.

The regional phylogenetic trees used in plant research are frequently obtained by pruning available megatrees that have been generated from the analysis of Sanger sequence data available in Genbank (Zanne et al. 2014; Smith and Brown 2018). However, the use of these megatrees has some limitations. First, their taxonomic sampling is only partial at the species level (ca. 9% of all vascular plant species in Zanne et al. (2014) and 23% in Smith and Brown (2018)) and unevenly representative across geographical regions and clades (Qian et al. 2021; Baker et al. 2022). Second, the low complementarity among the genes used to reconstruct these megatrees and their predominant plastid origin could bias the estimation of relationships and divergence time (Hinchliff and Smith 2014; Smith and Brown 2018). Although some evidence shows that these megatrees completed at the species level with priors derived from taxonomic classification might still be valid to detect broad‐scale patterns of phylogenetic diversity (Li et al. 2019; Qian and Jin 2021), other assessments at lower geographical scales have shown that partial or biased taxonomic sampling strongly impact the estimate of phylodiversity patterns within communities (Jantzen et al. 2019). Therefore, the phylogenetic framework provided by these megatrees might not always be suitable for exploring the processes of species diversification and assembly that contribute to the regional and local biodiversity patterns we observe today (e.g., Mishler et al. 2014; Cano et al. 2022; Smyčka et al. 2022).

With the development of high‐throughput DNA sequencing, approaches such as genome skimming and target sequence capture have contributed to the generation of more homogenous and comprehensive molecular datasets by extending DNA barcoding approaches to the genomic scale (Coissac et al. 2016; Dodsworth et al. 2019). Genome skimming, a method that takes advantage of shallow coverage shotgun sequencing, has emerged as a reliable and convenient method to easily obtain the whole plastome sequence even from historical herbarium samples (Alsos et al. 2020). For example, this method has been used to generate a regional phylogeny of all species of the arctic‐alpine flora (PhyloAlps and PhyloNorway projects; Alsos et al. 2024; Lavergne et al. 2025). Yet, several issues arise from using plastid loci for phylogenetic reconstruction, such as their low evolutionary rate and limited utility to resolve interspecific relationships (Naciri et al. 2012; Coissac et al. 2016; Caetano Wyler and Naciri 2016), their discordant evolution with species history caused by chloroplast capture consecutive to hybridization events (Rieseberg and Soltis 1991; Soltis and Kuzoff 1995; Tsitrone et al. 2003; Liu et al. 2020) and their relaxed evolution in non‐photosynthetic parasitic plants that may cause incorrect estimation of branch lengths (Wolfe et al. 1992). Genome skimming can also be applied to retrieve nuclear loci useful for phylogenomics such as ribosomal or low–copy genes (Liu et al. 2021; Meng et al. 2021; Pouchon and Boluda 2023; Zhang et al. 2024; Mo et al. 2025). However, such approach is generally more successful at low taxonomic levels (i.e., below families) than in large‐scale phylogenetic studies. Indeed, retrieving sets of conserved nuclear orthologs (e.g., BUSCO, Simão et al. 2015) across a wide range of taxonomical lineages from genome skimming data often requires massive sequencing effort, especially for plants with high genome sizes.

As an alternative method to genome skimming, target sequence capture has emerged as one of the most promising and cost‐efficient approaches to tackle the complexity of the nuclear genome by consistently gathering hundreds of nuclear loci even when herbarium specimens are used as source material (Brewer et al. 2019; Dodsworth et al. 2019). The Angiosperms353 baits set developed by Johnson et al. (2019) to target 353 single‐copy nuclear loci across all lineages of flowering plants proved to be a particularly powerful and versatile toolkit allowing to unravel the large‐scale evolutionary origins of angiosperms diversity (Zuntini et al. 2024), but also to resolve relationships and diversity patterns at much lower phylogenetic scales (Baker et al. 2021; McDonnell et al. 2021; Slimp et al. 2021; Stefani et al. 2025). The increasing use of Angiosperms353 is generating a new genomic resource for flowering plants that is today publicly accessible and covers 8000 (about 60%) angiosperm genera (Kew Tree of Life Explorer, Data release 3.0, April 2023; Baker et al. 2022; Zuntini et al. 2024). However, the Angiosperms353 dataset is still largely incomplete at the species level and, so far, the method has seldom been considered for regional biodiversity assessment (but see the GAP framework initiative; Simpson et al. 2024). Therefore, the suitability of Angiosperms353 for resolving both deep and shallow phylogenetic relationships among the different taxa composing a regional flora has yet to be evaluated.

Here, we developed a workflow based on the targeted gene capture of 353 nuclear loci with the universal probe set Angiosperms353 to assess the phylogenetic relationships among all species of a region's flora (Figure 1). To test our workflow, we constructed a species‐level phylogeny encompassing 92% of the 1098 indigenous angiosperm species of the Geneva canton flora in Switzerland. We carried out phylogenetic analyses of our nuclear dataset using concatenation, coalescent and time calibration approaches and compared the results with other megatrees available for angiosperms (Smith and Brown 2018; Zuntini et al. 2024). The nuclear Angiosperms353 species tree will also be compared with corresponding phylogenetic trees obtained from the analysis of plastid loci retrieved from off‐target reads, although we acknowledge that potential cyto‐nuclear discordance will not be analysed in a quantitative way in this paper. Here, we aimed to answer the following questions: (1) Is gene capture using Angiosperms353 suitable to generate genomic resources for regional floras? (2) Does Angiosperms353 improve phylogenetic resolution and yield more accurate age estimates than plastome datasets and existing large‐scale phylogenies for angiosperms? (3) Can phylogenomic analyses of regional floras provide insights to guide nomenclatural revisions?

**FIGURE 1:**
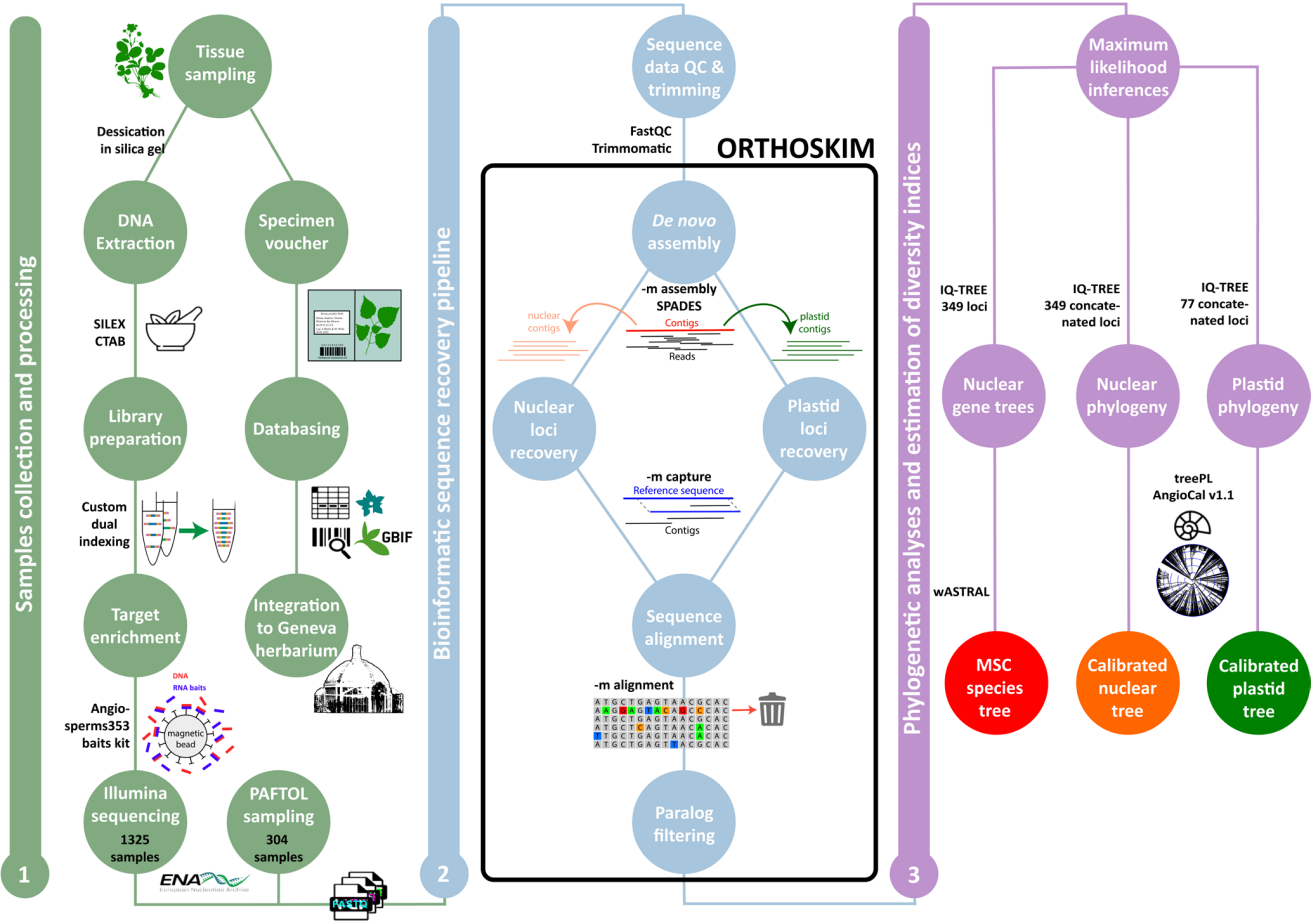
Graphic summary of the workflow used for this project, that goes from the collection of samples to the final phylogenetic output usable for phylogenetic diversity assessments.

## 2 Material and Methods

### 2.1 Study Area and Taxon Sampling

The angiosperm flora of the Geneva canton in southwestern Switzerland was selected for this study. The Geneva territory covers an area of 282.5 km^2^ that is part of the Swiss plateau biogeographic region (OFEV 2022). According to the latest Red List of Vascular Plants of the Geneva Canton, the area comprises 1098 indigenous angiosperms (Mombrial et al. 2020), representing 40.5% of the entire Swiss flora (Bornand et al. 2016). Our strategy consisted in sampling in the field most of these native species by taking advantage of expert knowledge on species preferences and extensive occurrence data deposited in the National data and information center on the Swiss flora (Info Flora, 277,748 observations recorded after 1995, with field accuracy ≤ 10 m). Tissue samples from species not found in the field (e.g., rare or regionally extinct species) were obtained from herbarium specimens stored in the Geneva herbarium (G). Field‐collected samples were immediately dried in silica gel to prevent DNA degradation. In parallel, a voucher specimen was collected in the field and deposited in the G herbarium. In total, one to three individuals per species were sampled. Information related to the specimens and DNA samples was recorded in the Geneva Herbarium Catalogue (https://collections.geneve.ch/jardinbotaniquegeneve/herbier/), the Tissue and DNA bank of the *Jardin Botanique de Genève* (JBGE‐DNA) and Info Flora using the FlorApp v. 3.0. We complemented our sampling strategy by mining the Plant and Fungal Trees of Life (PAFTOL) database (Data release 3.0, April 2023; Baker et al. 2022) for (i) species occurring in Geneva and Switzerland and (ii) species that could contribute to increasing the number of possible nodes available for fossil calibration. We used the taxonomic classification of the World Flora Online Plant List database (https://wfoplantlist.org, June 2024 snapshot). The information for samples collected and used in this work is available in Table S2.

### 2.2 DNA Extraction and Library Preparation

We extracted the total genomic DNA (gDNA) from 20 mg of silica‐dry tissue disrupted in a TissueLyser II (Qiagen, Venlo, the Netherlands) at a frequency of 30 Hz for 180 s. The cost‐efficient SILica matrix EXtraction (SILEX) protocol (Vilanova et al. 2020) was used to extract DNA from silica‐gel samples. For samples with low yield of DNA recovery (e.g., herbarium samples), we opted for a CTAB protocol (Doyle and Doyle 1987) that was modified as follows: 1% PVP‐40 was added to the CTAB buffer solution to reduce effects of phenolic compounds, 1/2 V:V ice cold isopropanol was used for nucleic acid precipitation instead of 2/3 V:V, and the precipitation was performed for 24 to 72 h at −20°C. Genomic DNA extracts were visualized in a 1% Agarose gel and quantified using a DeNovix DS‐11 FX spectrophotometer/fluorometer, with a DeNovix dsDNA High‐sensitivity kit (DeNovix, Washington, DE). DNA extracted from silica‐gel samples was sonicated for 75 up to 270 s at 25% intensity in a Qsonica Q800R3 Sonicator (Qsonica, Newtown, CT). The fragment size distribution for each sonicated sample was assessed on 1% Agarose gel using a Promega 100 bp DNA ladder (Promega, Madison, WI). We prepared libraries for each sample using 1000–3000 ng of gDNA in a total of 100 μL TE8 buffer, following a custom protocol described in De La Harpe et al. (2019). We proceeded to fragment size selection, using Serapure magnetic beads (Faircloth and Glenn 2011; Rohland and Reich 2012) and DNA ratios of 2.1 and 1.5, targeting fragment size ranges of 100–1000 bp and 300–1000 bp for herbarium and silica‐gel gDNA samples, respectively. Final DNA concentrations of libraries were checked using the DeNovix dsDNA High‐sensitivity kit, and their fragment size was verified using a BiOptic Qsep100 Bio‐Fragment Analyser with a standard S2 cartridge and visualized on the Q‐analyser software (BiOptic, New Taipei City, Taiwan).

### 2.3 Angiosperms353 Target‐Enrichment of Libraries and Sequencing

We prepared 56 pools of circa 25 libraries to reduce costs and maximize sequencing efficiency (Andermann et al. 2020). In order to minimize possible enrichment bias, pools of libraries were formed according to different criteria including, by order of importance: material type (silica‐gel/herbarium), taxonomic proximity, homogeneity of DNA fragment sizes and DNA concentration (Hale et al. 2020; Lee et al. 2021). We next performed target‐enrichment with the Angiosperms353 (Johnson et al. 2019) baits kit, following the myBaits standard protocol (Daicel Arbor Biosciences, Ann Arbor, MI), with a few modifications: the hybridization reaction was performed at 65°C for pools #1–4 or 61°C for pools #5–56 for 21 to 24 h. The post‐hybridization amplification was performed with the 2X KAPA HiFi HotStart Readymix and 10X Illumina Primer Mix provided with the KAPA HyperPrep kit, in duplicates for each pool to minimize amplification bias. The elongation time was set at 45 s (as recommended for fragments < 700 bp), and the number of cycles was set at 12 for silica‐gel and 14 for herbarium pools. The amplification product was purified with Serapure magnetic beads with 1.2 and 1.6 beads: DNA ratios for silica‐gel and herbarium sample pools, respectively. Duplicates were pooled together and quantified prior to analysing fragment sizes on an Agilent 2200 TapeStation (Agilent, Santa Clara, CA). Paired‐end sequencing (2*100 bp, except for pool 56 for which 2*250 bp paired‐end sequencing was used) was performed on an Illumina HiSeq 4000 (pools #1–4 and #6) or a NovaSeq 6000 (pools #5 and #7–56) sequencer at the iGE3 Genomics platform, University of Geneva, Switzerland for pools #1–25 and at the NGS platform, University of Bern, Switzerland for pools #26–55, respectively.

### 2.4 Bioinformatic Treatment of Sequence Data

Sequence data was processed according to the bioinformatic pipeline presented in Figure 1. The quality of the raw sequencing data was checked using FastQC v.0.12.0 (Andrews 2010) and MultiQC v.1.12 (Ewels et al. 2016). Trimmomatic v.0.38 (Bolger et al. 2014) was used to remove adapter sequences in samples with low molecular weight DNA, such as herbarium samples, and to filter out low‐quality reads, with the following parameters: a sliding window of 4, a mean quality score threshold cutoff of 18, a minimum quality score of 20 for leading and trailing bases, and a minimum read length of 20. We subsequently integrated the trimmed reads into the ORTHOSKIM v.1.6 pipeline (Pouchon et al. 2022) to recover target nuclear genes from the data along with plastid genes. We started by using the assembly mode (‐m assembly) with default parameters to perform a *de novo* assembly from raw reads, providing contigs that were then formatted (‐m format) and filtered (‐m cleaning) for contaminants, also with default parameters. Once we obtained the filtered contigs, we used the capture mode (‐m capture) to recover the Angiosperms353 genes set (‐t nucleus_aa), using a combination of the original Angiosperms353 reference file with the improved Mega353 fasta file (McLay et al. 2021) as reference sequences. During this step, one contig per gene was retained, corresponding to the candidate with the highest alignment score and sequence identity. This approach ensured the selection of the correct ortholog, if present, rather than paralogous sequences. The following parameters were used: (i) a minimal length of 90 nucleotides for captured sequences, (ii) a minimal coverage fraction of the reference set at 25%, (iii) a minimal contig k‐mer coverage of 3 and (iv) the minimal fraction of captured sequences covered by the longest ORF set at 80%. Regarding the 77 targeted plastid genes (‐t chloroplast_CDS, Table S4), we used the default reference file provided with ORTHOSKIM and the same parameters as the ones used for the nuclear genes.

The capture efficiency was measured by the number of recovered genes, their total sequence length and the ratio of reads on target for which the numerator is the number of mapped reads on target sequence and the denominator is the total number of reads. This ratio was obtained for each library by mapping reads on the closest reference sequences that were selected for each gene in ORTHOSKIM using BWA v.0.7.18 (Li and Durbin 2009). The ratio of reads mapped on target genes was then computed through the coverage function of samtools v. 1.19.2 (Danecek et al. 2021). We used the FullStats.py function in ORTHOSKIM to obtain the number of recovered genes and their total sequence length. To identify the factors influencing capture success, we tested for correlations between continuous variables (i.e., genomic DNA concentration, final library concentration, mean library fragment size and number of reads per sample) and capture efficiency by calculating either Pearson's or Spearman's correlation coefficient after checking for normality of data with the Shapiro–Wilk test. The effects of qualitative variables (i.e., data source, hybridization temperature, taxonomy at the ordinal level and material preservation type) on capture efficiency were tested using either ANOVA and Welch's two sample *t*‐test for parametric tests or Kruskal–Wallis/Wilcoxon rank sum tests for non‐parametric tests after checking for normality of data and equality of variances. Nuclear and plastid gene recovery heatmaps were generated in RStudio v. 2021.09.2 Build 382, using R v. 4.2.2 and the gplots v. 3.1.3 (Warnes et al. 2009) and RColorBrewer v. 1.1‐3 (Neuwirth 2022) packages.

### 2.5 Phylogenetic Analyses

We used the alignment mode (‐m alignment) of ORTHOSKIM to build single gene and concatenated alignment matrices independently for the plastid and the nuclear genes. The following parameters were used to identify and remove erroneous sequences or paralogs from single‐gene alignments, by using the FilterParalog.py function embedded in ORTHOSKIM v.1.6 and described in Lavergne et al. (2025): (i) the 100 bp generic level sliding window with a maximum of 20 polymorphic sites allowed, (ii) a minimal number of three taxa required to get a consensus sequence and (iii) a 150 bp family level sliding window with a maximum of 40 polymorphic sites allowed. The sliding window approach searches for gene sequences with hypervariable intragenic regions often indicating paralogous sequences. A computing error occurred during that step for four nuclear genes (numbered 4806, 5260, 5990 and 6320), which were removed from subsequent analyses.

We used IQ‐TREE v.2.2.2.6 (Minh et al. 2020) to infer maximum likelihood (ML) phylogenies from single‐genes and concatenated alignments for the nuclear dataset comprising 349 genes. We next performed a maximum likelihood phylogenetic analysis on the concatenation of 77 plastid coding sequences (CDS). Due to the large number of genes and samples, we used the default GTR + GAMMA substitution model and 1000 UltraFast Bootstrap (UFBoot) replicates to estimate branch supports for both datasets. Nuclear gene trees and alignments were trimmed from abnormally long branches detected by TreeShrink v.1.3.9 with default parameters (‐q 0.05; Mai and Mirarab 2018). This trimming step allows the removal of potential paralogous sequences that escaped the previous two filtering procedures during the capture and alignment steps. The resulting trimmed trees were used to estimate a coalescent‐based nuclear species tree with the weighted ASTRAL mode of ASTER v.1.16 (Zhang and Mirarab 2022). We selected the hybrid weighting mode, which uses both branch lengths and node support information to estimate local posterior probabilities (LPP), and set the minimum and maximum UFBoot support values at 20 and 100 respectively. As plastid genes are predicted to follow a concerted evolutionary history, no ASTRAL tree was computed for chloroplasts. We compared the trees obtained from different approaches and datasets using tanglegrams generated with the cophylo() function of the phytools v.2.5.2 R package (Revell 2012). For consistency, nodes with low bootstrap/LPP support (< 70%/0.7) were collapsed. Phylogenetic trees were visualized and annotated in iTOL v.5 (Letunic and Bork 2021).

### 2.6 Gene Orthology Inference

Gene orthology was further assessed using OrthoFinder v. 2.5.5 as an independent assessment of orthogroup consistency across the pipeline. We selected the Poaceae for this assessment because this family is well represented in our dataset (136 samples) and is characterized by a high incidence of polyploidy and hybrids, both of which are expected to increase the prevalence of paralogous genes (Stebbins 1985; Grass Phylogeny Working Group III 2025). Orthogroups were inferred from gene sets obtained after three successive processing stages: (i) capture step, (ii) the paralog filtering step and (iii) the TreeShrink step. To evaluate the effectiveness of our paralog‐filtering procedure, we recorded the number of inferred orthogroups for each Angiosperms353 locus and calculated the proportion of sequences assigned to the dominant orthogroup. These metrics were compared across the three filtering stages to assess changes in orthogroup composition throughout the pipline. To determine whether residual, unaccounted paralogs influenced species tree inference, we compared species trees generated from (i) the complete post‐TreeShrink Angiosperms353 alignments for Poaceae and (ii) a filtered dataset retaining only sequences assigned to the dominant OrthoFinder orthogroup for each Angiosperms353 locus. Gene trees were inferred with IQ‐TREE, using the GTR+G substitution model and 1000 UFboot replicates. Coalescent‐based species trees were subsequently estimated with ASTRAL using the same parameters as those applied to the complete dataset. The resulting species trees were compared using the cophylo() function implemented in the phytools package.

### 2.7 Divergence Time Estimation

We generated time‐calibrated nuclear and plastid phylogenetic trees under a penalized likelihood approach, as implemented in treePL (Smith and O'Meara 2012), which is computationally efficient for large datasets. A divergence time analysis Using Bayesian inference (e.g., BEAST; Heled and Drummond 2010) was not computationally feasible for a dataset of this magnitude. A single individual per species or subspecies was selected in the original ML trees according to the number of captured genes. Branch lengths information was derived from the concatenated ML tree which largely agrees with the ASTRAL species tree (see below). To calibrate our nuclear tree, we selected 45 fossils from the AngioCal v 1.1 dataset that could be used as age constraints in our phylogenetic tree (Table S7; Ramírez‐Barahona et al. 2020; Zuntini et al. 2024). When multiple fossil records could be attributed to the same node, the oldest fossil was selected for calibration. A maximum constraint was set at 154 My for the angiosperms crown node, corresponding to the lower boundary age estimate of the crown age for angiosperms (Sauquet et al. 2022; Zuntini et al. 2024). For the plastome phylogeny, a subset of 39 fossil calibration points was used for the dating. The *prime* command in treePL was first used to estimate the best optimization parameters for both nuclear (opt = 2, optad = 2, optcvad = 1) and plastid trees (opt = 2, optad = 2, optcvad = 0). A cross‐validation analysis was next conducted to estimate the optimal smoothing parameter (smoothing value of 0.1 for both trees) to generate the chronograms. To evaluate the robustness of node age estimates, we used the topology of the best ML tree as a constraint to generate 100 bootstrap trees and re‐estimate branch lengths. These bootstrap trees were dated using the same calibration points and parameters applied to the best nuclear tree. Bootstrap trees were summarized into a consensus tree using TreeAnnotator v 2.5.2 (Drummond and Rambaut 2007) to obtain confidence interval for each node. To assess the differences between our nuclear chronogram and the GBOTB chronogram available for angiosperms (Smith and Brown 2018), we first generated a subtree of the GBOTB chronogram with the V.PhyloMaker2 (Qian and Jin 2016; Jin and Qian 2022) R package, using the GBOTB.extended.TPL formatted tree. Then, we compared node ages estimates at the stem between the different chronograms, at the ordinal, family, generic and species taxonomic levels and statistically tested for correlation. R scripts used to perform these analyses are available on Zenodo under the DOI https://doi.org/10.5281/zenodo.22096205. All bioinformatic analyses were run on the high‐performance cluster of the University of Geneva (https://www.unige.ch/eresearch/en/services/hpc).

## 3 Results

### 3.1 Taxon Sampling

We collected 1628 plant samples representing 593 genera and 1316 species and subspecies. Most of these samples (1141, 70.1%) were collected in the field between March 2021 and October 2023. To complement this sampling, rare or locally extinct species were obtained from 183 herbarium specimens stored in the G herbarium (11.2%). We additionally incorporated to our dataset sequence data from 304 libraries (18.7%) mined from the PAFTOL database (Baker et al. 2022). Voucher information of these samples is summarized in Table S2.

### 3.2 Nuclear Loci Recovery

Sequencing yielded 4,733,171 paired‐end reads per library on average for the 1324 libraries that successfully passed through quality check. On average, 289 target genes and 152,819 bp of target sequence were recovered per sample (Table S3). The mean percentage of reads mapped on targets was 13.8%. We found that several factors significantly impact capture efficiency: taxonomy (measured at the ordinal level), material preservation type, data source and hybridization temperature during the capture step (Figure 2, Figure S1, Table S1). We found that Brassicales have the highest mean number of recovered genes (328; Figure 2a), whereas Asparagales have the lowest (219 genes). Likewise, Brassicales have the highest mean ratio of reads mapping on target (0.25; Figure S1a), and Alismatales have the lowest (0.02). The effect of taxonomy on the number of recovered genes can also be seen in the gene recovery heatmap (Figure S2). Regarding material preservation type, we found that samples dried in silica gel immediately after collection yield on average 298 target genes and 159,469 bp, and samples originating from herbarium specimens yield 207 target genes and 74,879 bp (Figure 2b). These values correspond to a 44% increase in gene recovery and a 113% increase in sequence length for silica‐dried specimens compared to herbarium specimens. No significant difference was found for the number of recovered genes and length of target sequence between samples sequenced in this study and in the PAFTOL project (Figure 2c). However, we found a higher ratio of reads on target for sequence generated in this study (0.15) than for those mined from the PAFTOL database (0.09, Figure S1c). Finally, we observed that target enrichment performed at 61°C increases the number of recovered genes (Figure 2d, mean = 291) and the length of target sequence (152,669 bp) compared to target enrichment performed at 65°C (276, 133,771 bp), and increases the ratio of reads mapping on target (Figure S1d, 0.15 vs. 0.12, only significant at *α* = 0.1). Additionally, we found a moderate positive correlation between the number of reads per sample and the number of recovered genes (*ρ* = 0.48, *p* value < 2.2e^−16^) and total sequence length (*ρ* = 0.45, *p* value < 2.2e^−16^). Other tested variables, mostly related to library preparation, showed significant but weak correlations (Table S1). Nuclear gene recovery statistics obtained from our analyses are available in Table S3. Raw FASTQ files for each sequenced individual are archived on the ENA database (project PRJEB88382).

**FIGURE 2:**
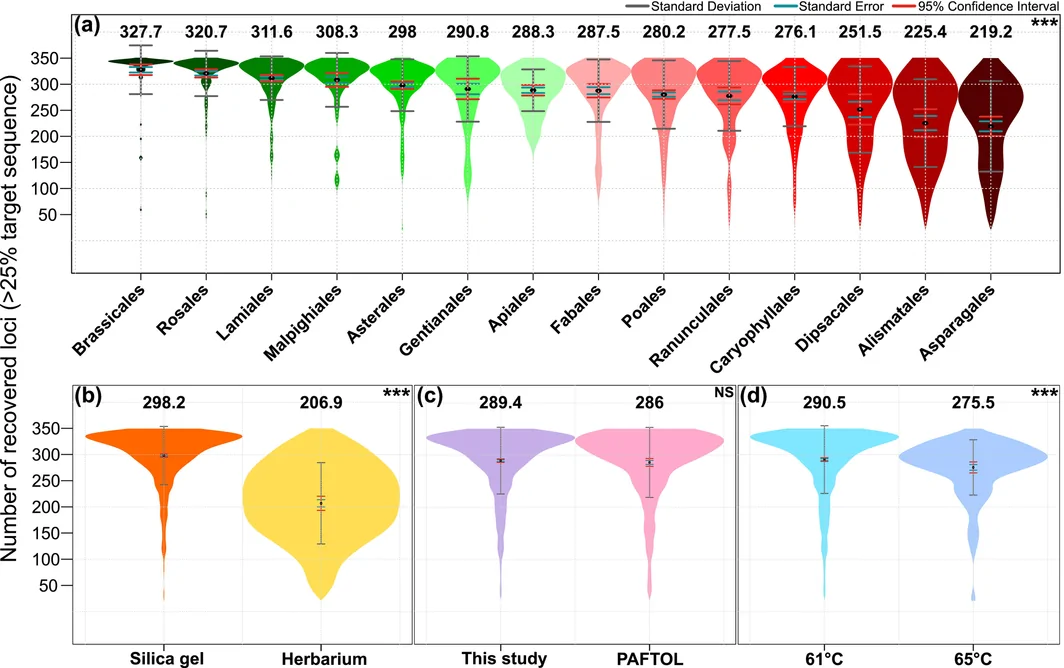
Violin plots showing how the number of targeted genes recovered per sample is impacted by factors like: (a) Taxonomy at the ordinal level (considering only orders with over 30 species). (b) Material preservation type. (c) Source of raw genetic data used to recover target genes. (d) Hybridization temperature used in the target enrichment step. Numbers in bold on top of the violin plots indicate the average for each category. Statistical significance is indicated on the top right corner of each plot (nominal alpha = 0.001 (***), NS = not significant).

### 3.3 Validation of Nuclear Gene Orthology Using Poaceae as a Representative Test Clade

Orthogroup consistency improved following paralog filtering during the alignment stage. The proportion of loci with > 90% of sequences assigned to the dominant orthogroup increased from 68.8% to 75.1%, whereas the proportion of loci exhibiting substantial orthogroup fragmentation (< 70% of sequences assigned to the dominant orthogroup) decreased from 9.4% to 7.2%. Subsequent TreeShrink filtering produced only a marginal additional increase in the proportion of loci with > 90% of sequences assigned to the dominant orthogroup (75.4%), while the proportions in the remaining categories remained unchanged.

Two species trees, each comprising 136 Poaceae taxa, were inferred: one from all Angiosperms 353 sequences retained by our workflow, and the other from the dataset containing only the dominant OrthoFinder orthogroup for each locus. Comparison of the resulting trees using a cophylogenetic plot revealed only four topological differences among the sampled taxa, three of which occurred within the genus *Bromus* (Figure S15). The fourth involved the placement of *Helictotrichon pubescens* , which was recovered as sister to Phleum in the tree inferred from the complete dataset, but as sister to the *Poa–Phleum* clade in the tree based solely on sequences assigned to dominant orthogroups. Overall, these results suggest that any residual paralogs remaining after our filtering procedure have only a limited effect on species‐tree topology in Poaceae. This finding also supports the robustness of our paralog‐filtering workflow and suggests that residual paralogy is unlikely to have substantially affected phylogenetic inference across the other taxonomic groups included in the Geneva regional flora.

### 3.4 Plastid Loci Recovery

An average of 54 plastid CDS per library was recovered from the off‐target reads (Figure S3). Among these loci, the standard DNA barcodes for plants *rbcL* and *matK* were retrieved in 72.4% and 71.8% of the species, respectively. In contrast to the nuclear dataset, we measured a significant effect of the data source on gene and total sequence recovery. Indeed, 58 CDS and 45,489 bp were recovered for this study's samples, whereas 36 loci and 21,732 bp were recovered for PAFTOL samples. No significant effect of the material preservation type was found for the number of recovered genes (*p* value = 0.34), but the total sequence length was significantly higher for silica‐gel samples (*p* value = 9.62e‐06). After the filtering step, 1531 samples were retained in the final dataset for the downstream phylogenetic analysis. Plastid gene recovery statistics obtained from our analyses are available in Table S3. Raw FASTQ files for each sequenced individual are archived on the ENA database (project PRJEB88382).

### 3.5 Phylogenetic Relationships Inferred From 353 Nuclear Genes

The alignment of the 349 nuclear genes obtained for 1628 plant samples consisted of a matrix of 175,252 bp, including 148,564 parsimony informative sites, with 23.4% of missing data. Both maximum likelihood and multi‐species coalescence inferences resulted in near complete phylogenetic trees of the regional angiosperm flora of the Geneva canton including 1316 species and subspecies distributed in 593 genera, 120 families and 43 orders. All orders and families were found monophyletic in the ASTRAL and ML trees. Samples from the same species were found monophyletic in both ASTRAL and ML trees for 87.1% of the species replicates.

Trees from ML and multi‐species coalescent (ASTRAL) analyses were overall well‐resolved and strongly supported, with an average UFBoot node support of 96% and 94% of all nodes confidently resolved for the ML tree (UFBoot > 70%; Figure 3) and an average local posterior probability (LPP) of 0.952 for the nodes of the ASTRAL tree (Figure S4). Both trees converged towards similar topologies at all taxonomic levels (Figure S6). Several conflicts were nonetheless observed such as (i) the position of Caryophyllales, nested between Santalales and Rosids in the ASTRAL tree but placed at the root of Pentapetalae in the ML tree, (ii) the relationship among orders in Malvids, (iii) the position of the Magnoliids clade that is sister to Ceratophyllales (UFBoot = 60%) in the ML tree, but sister to monocots in the ASTRAL tree (LPP = 1), (iv) Boraginales sister to Gentianales in the ML tree but sister to Solanales in the ASTRAL tree and (v) Poales sister to Commelinales in the ML tree but sister to Arecales in the ASTRAL tree. At lower taxonomic levels, the few conflicts between both topologies mainly involve weakly supported nodes within genera like *Rubus* (Rosaceae, Figure S7i), *Carex* (Cyperaceae, Figure S7d), among genera of the Orchidaceae family (Figure S7h), and in the Fabeae tribe of the Fabaceae family (Figure S7f).

**FIGURE 3:**
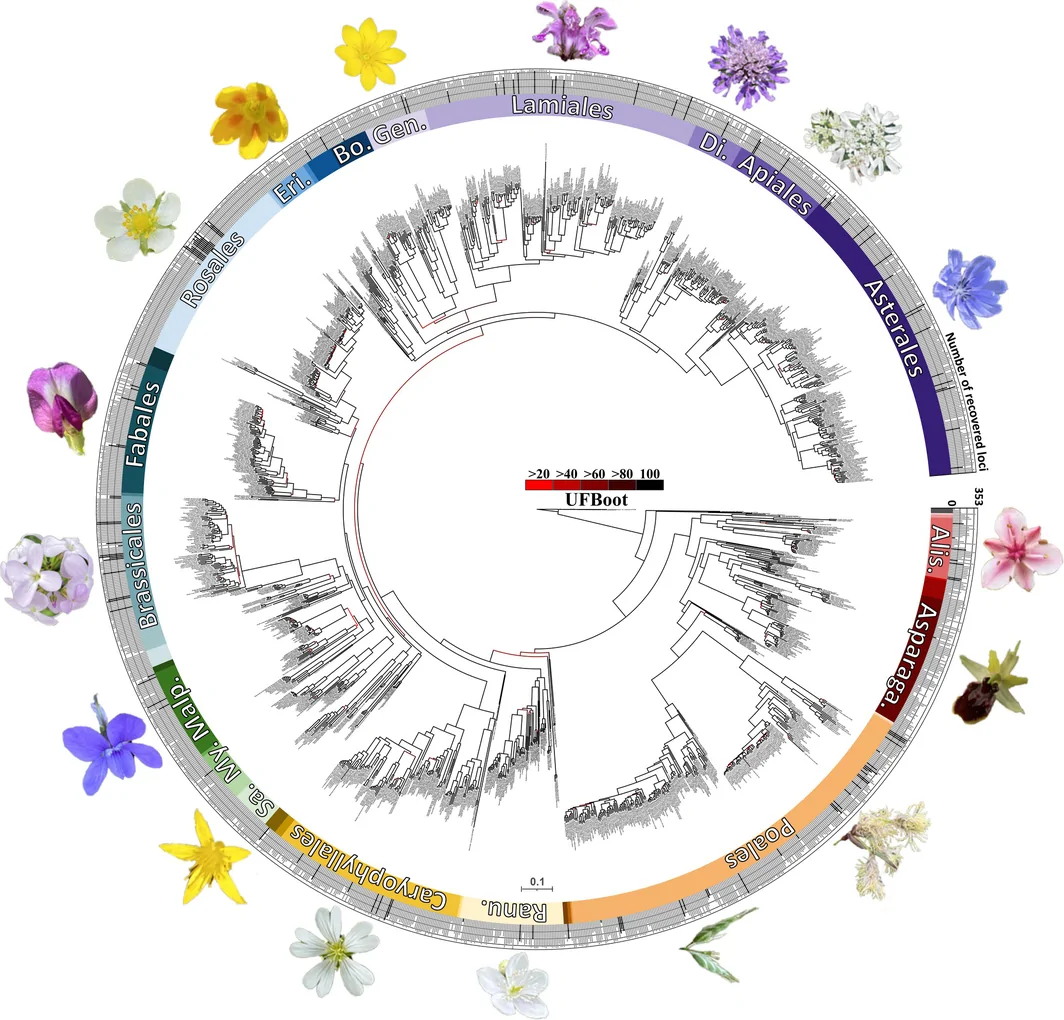
IQ‐TREE Maximum likelihood phylogeny for angiosperms of the Geneva flora based on the concatenation of 349 nuclear genes (175,252 bp) for 1628 taxa. Branches are coloured according to their UFBoot support, with red indicating low support and black high support. The coloured circle around the tree indicates boundaries between orders present in the phylogeny. The outer bar plots represent the number of nuclear loci captured for each sample, with light grey bars for silica‐gel samples and dark grey bars for herbarium samples. A selection of species representing different lineages of the Geneva flora are illustrated (Photo: S. Miche). The Newick tree file is available on Zenodo under the DOI https://doi.org/10.5281/zenodo.22096205.

### 3.6 Phylogenetic Relationships Inferred From 77 Plastid Genes

The alignment of 77 plastid genes obtained for 1531 plant samples produced a matrix of 56,342 bp, including 36,857 parsimony informative sites, with 31.1% of missing data. Analyses of this matrix using maximum likelihood resulted in a near‐complete well‐supported phylogenomic tree of the Geneva flora including 1261 species and subspecies distributed in 573 genera, 116 families and 41 orders (Figure S5). The average UFBoot support of nodes within the plastid tree is 89% and 85.1% of all nodes are confidently resolved in the ML tree (UFBoot > 70%). Samples from the same species are found monophyletic in the ML plastid tree in 81.6% of the cases.

### 3.7 Comparison Between Nuclear and Plastid Trees

We found that branch lengths of the nuclear tree are consistently longer than those of the plastid tree, as illustrated by the distributions of branch lengths in both trees across all major families comprising over 30 taxa (Figure S12). Very short branches (length < 0.001) are particularly frequent in the families Asteraceae, Rosaceae and Poaceae in the plastid tree but not in the nuclear tree (Figure S12). Moreover, the average number of informative sites per nuclear gene is 30.8% higher than that of plastid genes (Figure 4a), whereas node support is globally higher in the nuclear tree than in the plastid tree (Figure 4b). For example, certain families such as Alismataceae, Lythraceae or Scrophulariaceae have high average LPP in the coalescent species tree (0.99, 0.97 and 0.95 respectively), but low UFBoot support (74.3%, 76.2% and 77.5%) in the plastid tree (Figure 4b).

**FIGURE 4:**
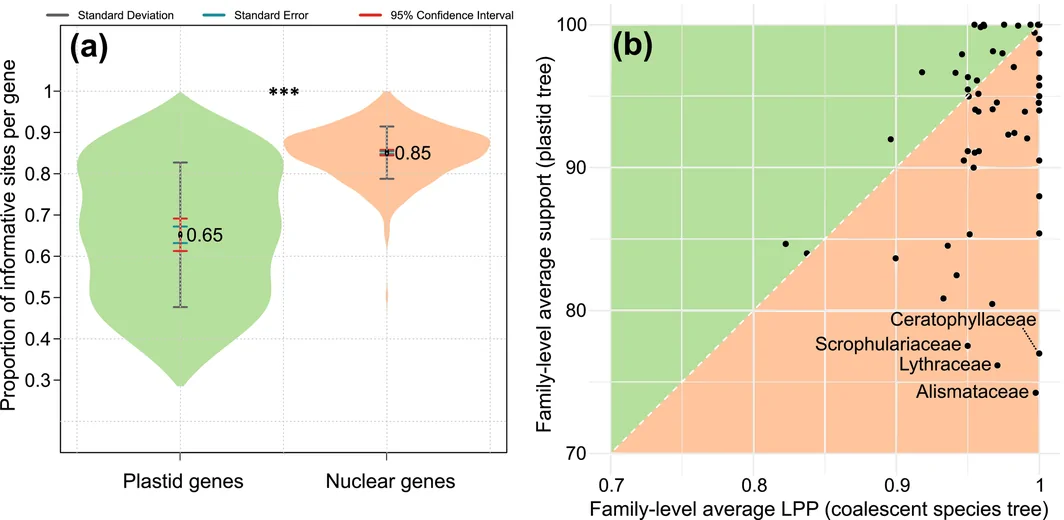
Differences in the number of informative characters and statistic support provided by the nuclear and plastid datasets used for reconstructing the phylogeny of the Geneva flora. (a) Violin plots display the ratio of informative sites per gene alignment for plastid (*N* = 77) and nuclear (*N* = 349) genes. Average values are indicated. (b) Plot comparing at the family level the average LPP values in the coalescent nuclear species tree (x‐axis) with the average UFBoot values in the plastid phylogeny (y‐axis). Dots in the bottom right triangle indicate families with better average support in the nuclear phylogeny, and dots in the top left triangle indicate families with better average support in the plastid phylogeny. Statistical significance is indicated (nominal alpha =  0.001 (***))

Topologies of the nuclear (concatenated) and plastid trees were globally congruent, yet several conflicts exist at the ordinal level (Figure S9): (i) the Magnoliids clade was sister to both monocots and eudicots in the plastid ML tree, and sister to Ceratophyllales in the nuclear ML tree, (ii) Caryophyllales was sister to the rest of the Pentapetalae clade in the nuclear tree but found embedded within Superasterids in the plastid tree, (iii) Santalales was placed at the root of Superasterids in the plastid tree and sister to Vitales in the nuclear tree where they form a monophyletic group at the root of Rosids, (iv) Aquifoliales was sister to Campanulids in the plastid tree and to Lamiids in the nuclear tree, (v) Lamiales was sister to the rest of Lamiids in the nuclear tree and to Boraginales in the plastid tree and (vi) within Rosids, orders Celastrales, Oxalidales and Malpighiales were placed within Fabids in the plastid tree but within Malvids in the nuclear tree. Moreover, the relationships between Rosids orders were differently organized between the nuclear and plastid trees (Figure S9). At the generic and species levels, both topologies were broadly concordant (Figure S10). However, species and genus swaps were observed in some families with poorly resolved lineages in the plastid tree (e.g., within Asteraceae, Brassicaceae, Cyperaceae, Fabaceae, Poaceae and Rosaceae, Figure S10). For example, in the Asteraceae genus swaps were observed in the Cichoreae tribe, and species swaps in the Carduineae subtribe which was monophyletic in the nuclear tree but paraphyletic in the plastid tree (Figure S10b).

### 3.8 Divergence Time Estimation

The time‐calibrated nuclear chronogram of the Geneva flora included 1300 unique taxa with 45 calibration points (Figure 5). We found that node age estimates obtained for this chronogram are reliable overall, as 98.6% of node age estimates fall within the confidence age intervals calculated from the 100 bootstrap replicates (Table S5). The plastid chronogram comprised 1235 taxa with 39 calibration points (Figure S11), and the GBOTB chronogram (Figure S13) included 1006 species that overlap with the species in our nuclear chronogram.

**FIGURE 5:**
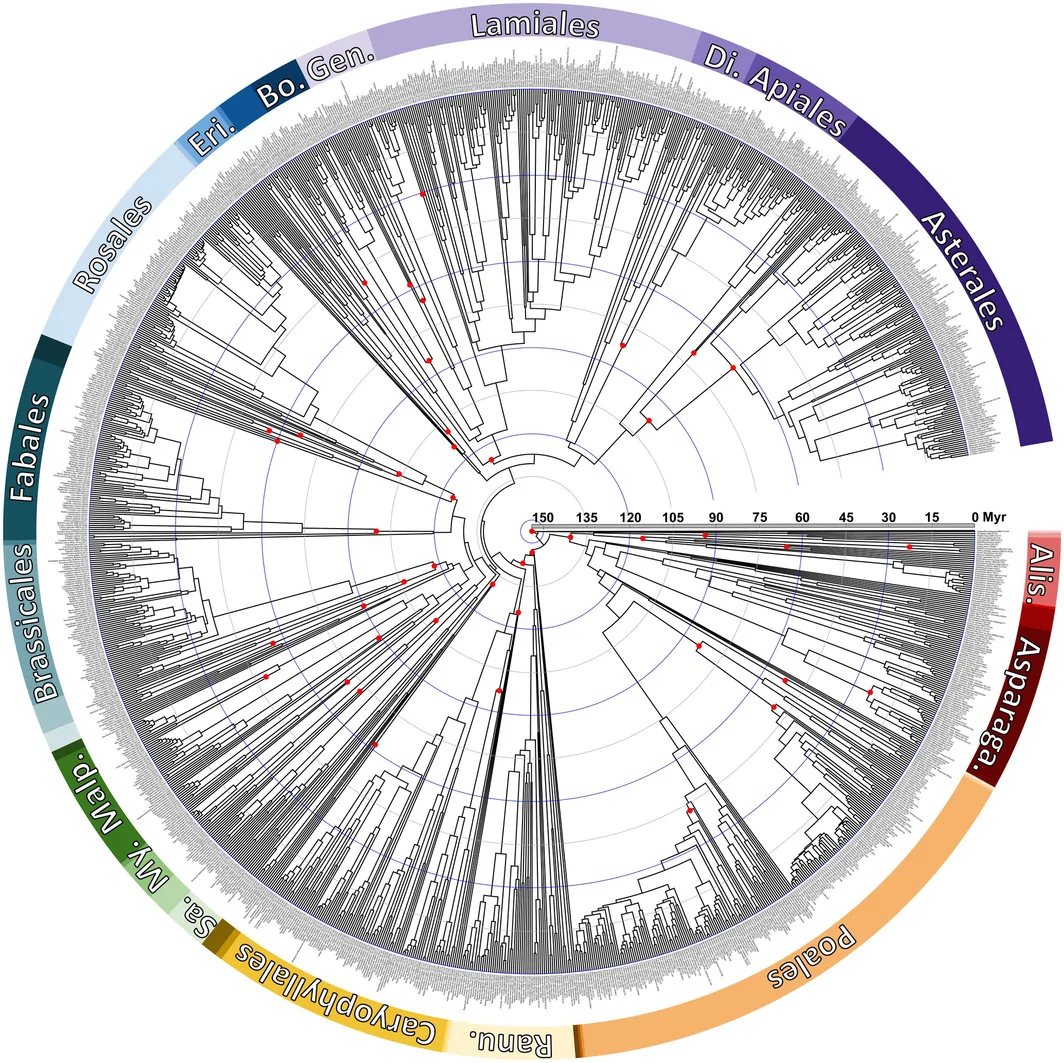
Nuclear chronogram of the Geneva flora containing 1300 taxa. A maximum constraint of 154 Myr was used at the root node and corresponds to the estimated upper boundary of the angiosperms crown age. Red dots at nodes indicate the placement of fossil calibrations extracted from the AngioCal v.1.1 dataset. The coloured circle drawn around the tree indicates boundaries between orders present in the phylogeny. The Newick tree file is available on Zenodo under the DOI https://doi.org/10.5281/zenodo.22096205.

The node age estimates between the nuclear and the plastid chronograms were highly similar and strongly correlated (*ρ* = 0.93, *p* value < 2.2e^−16^, Figure 6a). Node age estimates in our nuclear chronogram were also correlated with those of the GBOTB chronogram (*ρ* = 0.85, *p* value < 2.2e^−16^, Figure 6b), but our tree provided consistently older ages than in the GBOTB chronogram, with differences that increased with taxonomic ranks: +4.2 My on average for species, +8.4 My for genera, +18.8 My for families and +16.3 My for orders (Figure S14). The correlation between age estimates was lower at the species‐level (*ρ* = 0.71, *p* value < 2.2e^−16^) than at the generic, family and ordinal levels (*ρ* = 0.87, 0.85 and 0.88, *p* values < 2.2e^−16^, respectively). The analysis of standardized residuals allowed the identification of 84 outlier taxa that exhibit a disproportionate uncertainty in age estimates between chronograms. Half of species‐level outliers belonged to the Rosaceae family. For example, in the *Rubus*, *Rosa* and *Potentilla* genera, mean estimates of species ages in the GBOTB chronogram (respectively 27.8, 17.1 and 46.5 My) were much higher compared to the ages estimated in this study (means = 3.9, 4 and 7.7 My). Finally, we found a strong positive correlation (*ρ* = 0.85, *p* value < 2.2e^−16^) for the estimated stem ages of orders and families between our nuclear chronogram and the PAFTOL young tree (Figure 6c), although our age estimates were on average 14.8 My younger for orders and 8.1 My younger for families than in the PAFTOL chronogram.

**FIGURE 6:**
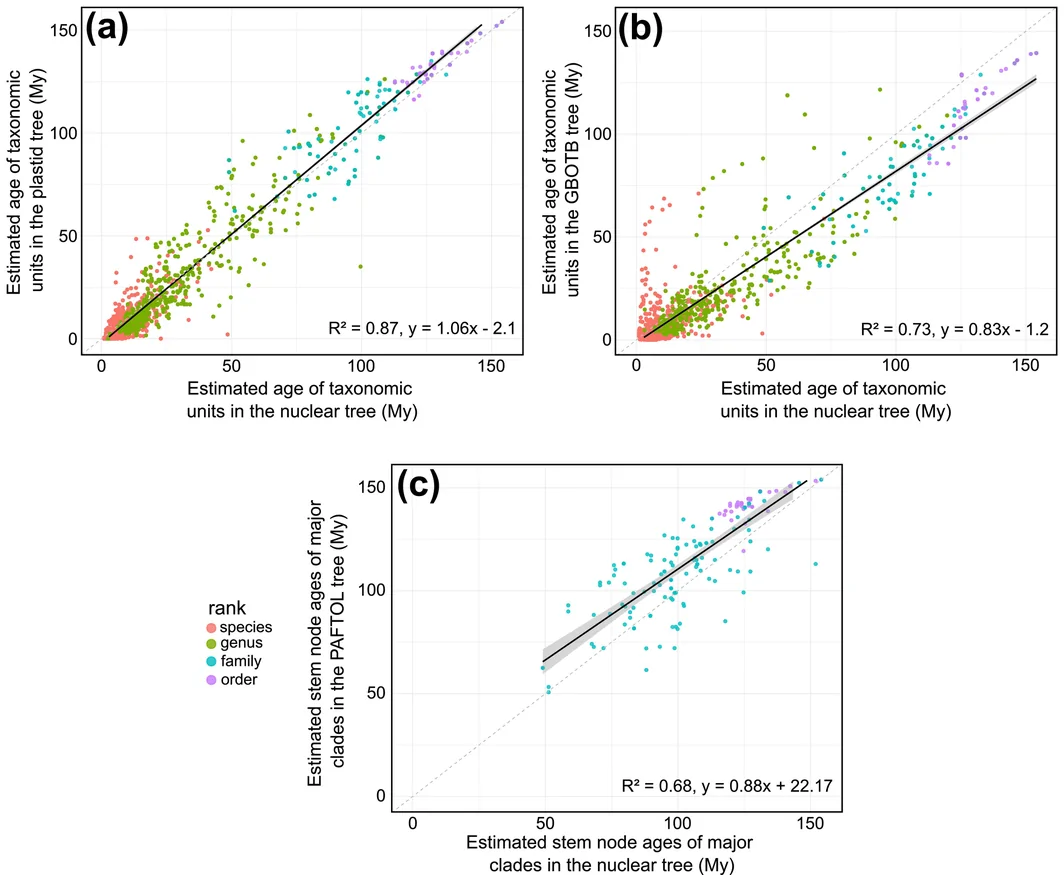
Comparisons of the estimated stem nodes ages (going to the most recent ancestor node) between the nuclear, plastid GBOTB and PAFTOL chronograms generated for the Geneva flora. (a) Comparison between the nuclear (x‐axis) and plastid (y‐axis) chronograms obtained in this study. (b) Comparison between the nuclear (x‐axis) and GBOTB (y‐axis) chronograms. (c) Comparison between the nuclear (x‐axis) and PAFTOL (young) (y‐axis) chronograms. Equations of the linear regressions and coefficients of determination are shown on the plots. Colours indicate the taxonomic rank of the node being either at the specific (pink), generic (green), family (blue) or ordinal (purple) levels.

## 4 Discussion

### 4.1 Methodological Advantages of Angiosperms353 and ORTHOSKIM Workflow to Generate Phylogenomic Resources at the Regional Scale

Our results show the usefulness of generating Angiosperms353 sequences data for a large number of species composing a regional flora. Despite the narrow geographical scale of our study, focused on the Geneva canton, sampled taxa represent 44.6% of all native species in Switzerland (Bornand et al. 2016), providing a valuable resource to investigate systematic and biodiversity questions at the national scale. Our sampling conducted in the field and from herbarium specimens also makes publicly available original sequences data for 902 taxa missing from the PAFTOL data release (data release 3.0, April 2023; Baker et al. 2022) and 308 species lacking from the GBOTB tree of life (Smith and Brown 2018), demonstrating the success of our regional‐focused sampling strategy to complete the existing resource used to infer the angiosperms tree of life.

The mean number of nuclear genes we recovered per sample (298 for silica‐dry and 206 for herbarium samples) is comparable to those reported in other recent studies with broad taxon sampling (Brewer et al. 2019; Antonelli et al. 2021; Maurin et al. 2021; Thomas et al. 2021). We also found that 44% more genes and 113% longer sequences on average were recovered from silica‐dry samples compared to herbarium samples, highlighting the importance of samples' preservation methods in the yield of usable sequence data. We found that the capture efficiency of Angiosperms353 varies among the 42 orders sampled in this study (Figure 2), with a notably low efficiency for the Asparagales and Alismatales as previously reported (Figure 2; Haigh et al. 2023; Simpson et al. 2024). These results confirm a taxonomical bias in the capture efficiency of Angiosperms353 (Brewer et al. 2019; Antonelli et al. 2021) and emphasize the necessity to improve the Angiosperms353 baits kit by enhancing the representativity of references used for designing baits in certain lineages.

Despite a 31% decrease in the number of recovered nuclear genes for herbarium samples compared to fresh silica‐dried samples, we successfully generated sequences for 183 herbarium samples, including 79 endangered and 104 regionally extinct species that have not been observed in the region since the first exhaustive inventory of the Geneva flora in 1966 (Weber 1966; Mombrial et al. 2020). For example, DNA extracted from a *Schoenoplectiella supina* (L.) Lye specimen collected in 1839 yielded 191 target genes and 53,514 bp. Once abundantly distributed on the shores of Swiss lakes, this species was last seen in 1861 in the Geneva canton (Mombrial et al. 2020) and is now critically endangered or potentially extinct in Switzerland (Juillerat et al. 2017). Generating sequence data for such species that have been extirpated from the regional flora opens the possibility to monitor the loss of evolutionary diversity through time and to detect phylogenetic signals of extinction and turnover at the regional scale (Winter et al. 2009; Molina‐Venegas et al. 2020).

The combination of a single laboratory workflow with a unified bioinformatic pipeline (Figure 1) enables the efficient retrieval of both targeted nuclear and plastid datasets, demonstrating the efficacy and reliability of ORTHOSKIM for applications extending beyond genome skimming (Pouchon et al. 2022). Using this strategy to analyse our off‐target reads (representing on average 86% of the raw reads) allowed the recovery of a substantial proportion of plastid coding sequences, with an average of 68% of all plastid CDS retrieved across our samples. This result corroborates previous studies showing that plastid genomic regions can be consistently recovered from sequence data generated through Angiosperms353 target enrichment (Antonelli et al. 2021; Lee et al. 2021). We found no significant difference in the number of recovered genes between herbarium and silica‐gel samples, supporting earlier findings that the type of starting material has little impact on sequence recovery rate when plastid CDS are targeted (Alsos et al. 2020).

Plastome data retrieved from the off‐target reads may also complement existing other plastid genome reference datasets, such as PhyloAlps and PhyloNorway, which are used to infer regional phylogenies, or to identify ancient DNA from environmental samples (Alsos et al. 2020, 2024; Lavergne et al. 2025). More broadly, our bioinformatic pipeline could be readily extended to recover widely used loci for plant phylogenetics (e.g., ITS) and DNA barcoding (e.g., *rbc*L, *mat*K, *trn*L), thereby contributing to the enrichment of sequences data deposited in Genbank and in the Barcode of life Database (BOLD, Ratnasingham and Hebert 2007).

### 4.2 Nuclear Versus Plastid Phylogenomic Comparisons

Phylogenetic relationships within families are consistently well supported in the nuclear tree, even in cases where family‐level UFBoot support is low in the plastid tree (Figure 4b). The nuclear dataset also provides a higher number of informative sites per gene than plastid genes (Figure 4a), reflecting the faster evolutionary rates of nuclear loci. This characteristic of the nuclear genome represents an advantage for phylogenetic inference at shallow taxonomic levels or among rapidly diverging lineages, compared with plastomes.

The nuclear tree has better resolution than the plastid tree, which can be explained by longer average branch lengths (Figure S12). Longer branch lengths in the nuclear tree likely result from the greater number of informative sites per gene (Figure 4a). Greater informativeness reflects the faster evolutionary rates of nuclear loci and represents an advantage for phylogenetic inference at shallow taxonomic levels or among rapidly diverging lineages, compared to plastid loci (Small et al. 2004).

Despite the overall high congruence between the nuclear and plastid topologies, we identified several cyto‐nuclear conflicts occurring at different taxonomic scales. A notable example of ancient cyto‐nuclear discordance concerns the placement of Magnoliids (Piperales and Laurales), which appear sister to both monocots and eudicots in the plastid phylogeny, but as sister to eudicots in the nuclear concatenation tree (Figure S9a). This discordance has been attributed to ancient hybridization events associated with the early diversification of angiosperms (Zeng et al. 2014; Yang et al. 2020; Guo et al. 2021). Similarly, the unstable placement of Caryophyllales across our different phylogenetic inferences could reflect a complex history involving multiple whole genome duplication events (Walker et al. 2018; One Thousand Plant Transcriptomes Initiative 2019). Indeed, it has been shown that this placement cannot be confidently resolved using Angiosperms353 data alone (Zuntini et al. 2024).

Cyto‐nuclear discordances are more frequent at lower taxonomic ranks, particularly at the generic and infrageneric levels. For example, within Poaceae, the relative positions of the *Catabrosa*, *Festuca*, *Holcus*, *Lolium*, *Milium* or *Poa* differ between the nuclear and the plastid trees (Figure S10j). Such incongruences may reflect reticulation events that have repeatedly occurred in Poaceae (Grass Phylogeny Working Group III 2025). However, these discordant relationships receive weak support in the plastid phylogeny (< 70% UFBoot), and their significance requires further investigation. At the infrageneric level, pronounced topological discordance is observed in several genera, including *Carex*, *Rosa*, *Rubus* and *Salix* (Figure S10). These patterns may result from rapid and recent radiations within these lineages combined with processes such as hybridization, chloroplast capture and allele surfing associated with repeated range expansions (Currat et al. 2008; Martín‐Bravo et al. 2019; Naciri and Linder 2020). By simultaneously generating both nuclear and plastid genomic resources, our workflow provides a valuable framework for further exploring these processes. In particular, it enables the investigation of evolutionary events such as chloroplast capture or plastid haplotype sharing among closely related species co‐occurring within the same region (Naciri and Linder 2015).

Age estimates in our nuclear chronograms were strongly correlated with those from our plastid chronogram (Figure 6a, *ρ* = 0.93), as well as with age estimates from large‐scale published chronograms including the GBOTB tree (Figure 6b, *ρ* = 0.85, *p* value < 2.2e^−16^; Smith and Brown 2018) and the PAFTOL young tree (Figure 6c, *ρ* = 0.85, *p* value < 2.2e^−16^; Zuntini et al. 2024). These results indicate that molecular dating based on nuclear and plastid genomes is congruent and globally consistent with large‐scale chronograms widely used to study angiosperms diversity. However, our nuclear calibration yielded younger age estimates compared to the PAFTOL tree, likely due to the smaller number of calibration points and the sparser taxonomic sampling in our dataset (Sauquet et al. 2012; Magallón et al. 2015). In contrast, our age estimates were generally older than those of the GBOTB tree, reflecting differences in the calibration strategy and an older maximum constraint applied to the angiosperm crown age in our analyses (154 My) compared with the GBOTB analyses (139 My) (Smith and Brown 2018). At the species‐level, age estimates in our chronogram were less strongly correlated with those of the GBOTB chronogram (*ρ* = 0.71) than at higher taxonomical ranks (*ρ* = 0.85–0.88, Figure 6b), suggesting potential biases in the species‐level age estimates of the GBOTB chronogram. Notably, Rosaceae showed the highest proportion of age outliers (50%) in the linear regression comparing our chronogram with the GBOTB chronograms. For instance, our age estimates for *Rubus* (mean = 3.7 My) are consistent with the proposed Pliocene/Pleistocene origin of this genus in European (Carter et al. 2019), whereas the GBOTB estimates are considerably older (mean = 27.8 My). Such overestimation of clade ages in the GBOTB megatree could bias phylogenetic diversity assessment in certain floristic assemblages. Overall, our time‐calibration strategy provides a robust regional evolutionary framework that integrates and reliably estimates the age of all taxa, including at the species‐level.

### 4.3 Systematic and Conservation Implications

We used the newly generated Angiosperms353 dataset for 1628 plant samples to infer a comprehensive regional maximum likelihood phylogeny of the Geneva canton flora, that is highly supported (mean UFBoot = 96%) and resolved at all taxonomic levels. Its topology is highly congruent with the ASTRAL tree (Figures S6 and S7), despite few conflicts found at nodes involving short branches within few species rich genera, for example, *Carex*, *Lathyrus*, *Rubus*, with recent and/or complex evolutionary histories comprising frequent hybridization events, apomictic reproduction or incomplete lineage sorting arising from rapid diversification events or stochasticity (Naciri and Linder 2015; Caetano Wyler and Naciri 2016; *Carex*: Gebauer et al. 2014; The Global Carex Group et al. 2016; *Lathyrus*: Schaefer et al. 2012; *Rubus*: Sochor et al. 2015; Carter et al. 2019). Conflicting nodes between the concatenation and ASTRAL trees also exist at the ordinal level where uncertainty could be linked to incomplete lineage sorting due to ancestral genome duplication events and/or horizontal gene flow that occurred during the diversification of extant angiosperms orders (Naciri and Linder 2020; Stull et al. 2020; Zuntini et al. 2024). Overall, we obtained a higher topological congruence between the nuclear concatenated ML and ASTRAL trees (Figure S7) than between the nuclear and plastome trees (Figure S10) highlighting the fact that the source genomes used to infer phylogenies have a higher impact on tree topologies than the method used to reconstruct phylogenies.

We found a high topological congruence between our nuclear tree and the recently published generic‐level tree of angiosperms (Figure S8; Zuntini et al. 2024). The few conflicts observed mainly concern relationships at the ordinal level that are poorly supported in either tree (e.g., Ceratophyllales; Figure S8), or that could result from the absence of key intermediary clades in our ASTRAL tree potentially causing incorrect sister relationships (e.g., because of long branch attraction). At the generic and infra‐generic levels, our topology largely agrees with the WFO nomenclature but also confirms the paraphyly of several problematic clades that have been previously revised based on Sanger sequence data and that still await formal taxonomic revisions. For example, the paraphyly of the *Campanula* genus observed in our tree, with a clade comprising *Legousia* and *Phyteuma* as sister to *Campanula persicifolia* , had already been reported in several previous family‐level phylogenies based on a more extensive taxonomic sampling (Mansion et al. 2012; Crowl et al. 2014; Xu and Hong 2021). Our results also support the paraphyly of the genus *Vicia* caused by the position of *V. hirsuta* sister to the *Vicia‐Lathyrus* clade (Figure S7f) and the paraphyly of *Arabis* and *Draba* (Figure S7c) that have been previously reported without reclassification (Schaefer et al. 2012; Karl and Koch 2013). Likewise, our results fully agree with the many phylogenetic‐based revisions of European taxa that have been proposed for the *Chenopodium* s.l. (Fuentes‐Bazan et al. 2012), *Sorbus* s.l. (Figure S7l; Lo and Donoghue 2012), *Festuca*/*Lolium* (Figure S7j; Charmet et al. 1997; Gaut et al. 2000; Torrecilla and Catalán 2002), *Polygonum/Persicaria* (Kim and Donoghue 2008), *Anacamptis/Orchis* (Figure S7h; Bateman et al. 2003; Inda et al. 2012), among others. The extensive congruence between our results and current systematic knowledge across the angiosperm tree of life indicates that our phylogenomic resource provides a sound and unified taxonomic backbone that could serve as reference for evaluating taxonomic status and justifying nomenclatural changes within a specific flora. Although we agree that clade‐based systematics is the most appropriate approach to tackle taxonomic issues, we advocate that our phylogenetic resource based on a uniform set of genes and an homogenous sampling area could be easily expanded to evaluate the taxonomic circumscription of geographically restricted taxa or to guide nomenclatural updates of national or regional floras at the generic and infrageneric levels (Juillerat et al. 2017).

Our regional phylogeny also adds an evolutionary framework to existing biodiversity resources deriving from monitoring programs and Red Lists assessment available for the Geneva canton (Mombrial et al. 2020; Honeck et al. 2020) and Switzerland (BDM Coordination Office 2014; Bornand et al. 2016). It opens perspectives for conservation measures that integrate evolutionary history (Forest et al. 2007, 2018; Pipins et al. 2024), and provides a better understanding of the phylogenetic structure of species assemblages at the local or regional scale (Knapp et al. 2008; Winter et al. 2009; Thornhill et al. 2017; Spalink et al. 2018; Saladin et al. 2020).

## 5 Conclusion

Here, we describe a workflow that takes advantage of target enrichment to resolve the phylogenetic relationships at the species level for a regional flora, using a set of 353 nuclear genes common to Angiosperms (Figure 1). Following this workflow, we successfully generated a comprehensive time‐calibrated phylogeny of the Geneva flora that is more robust than trees obtained from plastomes or by pruning megatrees available for angiosperms (e.g., using PhyloMaker; Qian and Jin 2016). We highlighted that the Angiosperms353 genes have the potential to solve most of the taxonomic conflicts related to genera circumscription previously reported in the literature. Finally, we explored the combinatory potential of Angiosperms353 by completing our dataset with samples mined from the PAFTOL database (Baker et al. 2022). This scalability paves the way to future expansions of the dataset to encompass entire flora in Switzerland or in any other regions around the world.

## Author Contributions

M.P., S.M. and N.W. designed the study. S.M., M.W. and M.P. contributed data. S.M. analysed the data with substantial support from C.P., C.C. and A.M. All authors contributed to the writing and approved the final manuscript.

## Funding

This research received funding from the Plant Sciences department of the University of Geneva, the Geneva Botanic Garden, the Dr. Joachim de Giacomi Legacy funds of the Swiss Academy of Sciences (SCNAT), the Swiss Systematics Society, the *Office Cantonal de l'Agriculture et de la Nature de Genève* (OCAN) and the Swiss Federal Office for Environment (FOEN) through the convention‐programme *RPT LPN 2020–2024—Domaine protection de la nature*.

## Ethics Statement

Our work follows all ethical and research integrity standards.

## Conflicts of Interest

The authors declare no conflicts of interest.

## Supporting information


**Figure S1:** Violin plots comparing the ratio of reads mapped on target compared to the total number of reads for all samples according to several factors: (a) Taxonomy at the ordinal level for orders comprising over 30 species (samples of this study only). (b) Material preservation type. (c) Source of raw genetic data used to recover target genes. (d) Hybridization temperature used in the target enrichment step (samples of this study only). Numbers in bold on top of the violin plots indicate the average for each category. Statistical significance is indicated on the top right corner of each plot (nominal alpha = 0.1 (.), 0.05 (*), 0.01 (**), 0.001 (***), ns = not significant).
**Figure S2:** Heatmap of gene recovery efficiency, in which rows represent all 1628 samples and columns represent the 353 target genes hierarchically clustered into three groups with different gene recovery efficiencies. A darker colour indicates that a higher percentage of the target gene has been recovered. On the left side of the heatmap, the cladogram represents the phylogenetic position of samples included in the heatmap. The coloured bar drawn next to the cladogram indicates boundaries between orders present in the phylogeny (see Figure 3 for more details). On the right side of the heatmap, barplots indicate the number of genes recovered per sample, with silicagel preserved samples in green and herbarium preserved samples in orange.
**Figure S3:** Heatmap of gene recovery efficiency, in which rows represent all 1628 samples, ordered according to their phylogenetic position, and columns represent the 79 plastid genes.
**Figure S4:** Weighted‐ASTRAL multi‐species coalescent phylogeny of the Geneva flora from 349 nuclear gene trees with the hybrid‐weighting mode. The coloured circle around the phylogeny delimits all 41 orders of angiosperms plus the gymnosperms outgroup. The coloured circle drawn around the tree indicates boundaries between orders present in the phylogeny. The outer barplots represent the number of plastid loci retrieved for each sample. Coloured branches represent the local posterior probability (LPP) of internal nodes, red meaning low LPP and black high LPP. The Newick tree file is available on Zenodo under the DOI https://doi.org/10.5281/zenodo.22096205.
**Figure S5:** IQ‐TREE Maximum likelihood phylogeny for angiosperms of the Geneva flora based on the concatenation of 77 plastid genes for 1532 taxa. The coloured circle around the phylogeny delimits all 41 orders of angiosperms plus the gymnosperms outgroup. The coloured circle drawn around the tree indicates boundaries between orders present in the phylogeny. The outer barplots represent the number of plastid loci retrieved for each sample. This phylogeny was inferred using a concatenated alignment of 56,342 positions, and the statistical support of nodes was inferred with 1000 Ultrafast Bootstrap (UFBoot) replicates. Coloured branches indicate the support of internal nodes, red meaning low support and black high support. The Newick tree file is available on Zenodo under the DOI https://doi.org/10.5281/zenodo.22096205.
**Figure S6:** Cophylogenetic plots of major clades between this study's ML (left) and ASTRAL(right) nuclear phylogenies. (a) Order level comparison. (b) Family level comparison. Families comprising more than 30 samples are highlighted in red and detailed in Figure S7.
**Figure S7:** Species‐level cophylogenetic plots of major families (> 30 samples) between the ML (left) and ASTRAL (right) nuclear phylogenies. (a) Apiaceae. (b) Asteraceae. (c) Brassicaceae. (d) Caryophyllaceae. (e) Cyperaceae. (f) Fabaceae. (g) Lamiaceae. (h) Orchidaceae. (i) Plantaginaceae. (j) Poaceae. (k) Ranunculaceae. (l) Rosaceae. Nodes with low UFBoot support (< 70) or LPP (< 0.7) are resolved as polytomies.
**Figure S8:** Cophylogenetic plots of major clades between this study's ML nuclear (left) and PAFTOL young (right) phylogenies. (a) Ordinal level comparison. (b) Family level comparison.
**Figure S9:** Cophylogenetic plots of major clades between this study's ML nuclear (left) and plastid (right) phylogenies. (a) Ordinal level comparison. (b) Family level comparison. Families comprising more than 30 samples are highlighted in red and detailed in Figure S10.
**Figure S10:** Species‐level cophylogenetic plots of major families (> 30 samples) between the nuclear (left) and plastid (right) maximum likelihood phylogenies. (a) Apiaceae. (b) Asteraceae. (c) Brassicaceae. (d) Caryophyllaceae. (e) Cyperaceae. (f) Fabaceae. (g) Lamiaceae. (h) Orchidaceae. (i) Plantaginaceae. (j) Poaceae. (k) Ranunculaceae. (l) Rosaceae. Nodes with low UFBoot support (< 70) are resolved as polytomies.
**Figure S11:** Plastid chronogram of the Geneva flora containing 1235 taxa. A maximum constraint of 154 Myr was used at the root node and corresponds to the estimated upper boundary of the angiosperms crown age. Red dots at nodes indicate the placement of fossil calibrations extracted from the AngioCal v.1.1 dataset. The coloured circle drawn around the tree indicates boundaries between orders present in the phylogeny. The Newick tree file is available on Zenodo under the DOI https://doi.org/10.5281/zenodo.22096205.
**Figure S12:** Barplots of the distribution of branch lengths in the nuclear (orange) and the plastid (green) trees for families that comprise > 30 samples.
**Figure S13:** GBOTB chronogram of the Geneva flora generated by pruning the GBOTB.extended.TPL backbone available in PhyloMaker. Time scale represents the estimated age of each taxa in the chronogram.
**Figure S14:** Violin plots of the difference in estimated ages of taxonomic units between the nuclear chronogram and the plastid chronogram (top); and between the nuclear chronogram and the GBOTB chronogram (bottom).
**Figure S15:** Cophylogenetic plots of the ASTRAL species trees of 136 Poaceae accessions inferred from (left) the dataset containing only the dominant OrthoFinder orthogroup for each Angiosperms353 locus, and (right) from all Angiosperms353 sequences retained by our workflow. Differences of topology are highlighted in red.
**Table S1:** Effects of factors and variables on capture efficiency. For factor effects, *p* value of the appropriate statistical test is indicated, for correlations with variables, the Spearman rho value is indicated in the first row, and the *p* value for the statistical significance of the correlation is indicated in the second row. Symbols in parenthesis indicate the statistical significance at the nominal alpha = 0.1 (.), 0.05 (*), 0.01 (**), 0.001 (***) levels, (ns) being not significant (two‐tailed tests). Detailed results for each sample are available in Table S3.
**Table S4:** List of plastid genes recovered with ORTHOSKIM that were used in the phylogenetic inference.

**Table S2:** Samples included in our nuclear and plastid dataset of the Geneva angiosperm flora, including voucher information and SRA numbers from ENA.

**Table S3:** Recovery success and sequence length of the Angiosperms353‐targeted loci.

**Table S5:** ML phylogeny nodes for which age calibration falls outside the confidence interval obtained with calibration of 100 bootstrap trees with independent branch length.

**Table S6:** Proportion of sequences in dominant orthogroup per Angiosperms353 gene after each of the filtering step.

**Table S7:** List of fossils selected for calibration of the regional tree of angiosperms.

## Data Availability

Raw sequence reads for the NGS Illumina runs were deposited in the European Nucleotide Archive (ENA) under Bioproject no. PRJEB88382. Sequences and metadata of the plant samples sequenced in this study are available in Table S2 and partially available in the Kew Tree of Life Explorer 4.0 (https://treeoflife.kew.org/). Images and information related to each voucher specimen are publicly available in the Geneva Herbarium Catalogue (https://collections.geneve.ch/jardinbotaniquegeneve/herbier/). The tree files and source code are available at Zenodo under the https://doi.org/10.5281/zenodo.22096205. Benefits from this research are shared through open access to our workflow and data.

## References

[men70202-bib-0001] Allen, J. M. , C. C. Germain‐Aubrey , N. Barve , et al. 2019. “Spatial Phylogenetics of Florida Vascular Plants: The Effects of Calibration and Uncertainty on Diversity Estimates.” iScience 11: 57–70. 10.1016/j.isci.2018.12.002 PMC6308250 30590251

[men70202-bib-0002] Alsos, I. G. , V. Boussange , D. P. Rijal , et al. 2024. “Using Ancient Sedimentary DNA to Forecast Ecosystem Trajectories Under Climate Change.” Philosophical Transactions of the Royal Society, B: Biological Sciences 379: 20230017. 10.1098/rstb.2023.0017 PMC10999269 38583481

[men70202-bib-0003] Alsos, I. G. , S. Lavergne , M. K. F. Merkel , et al. 2020. “The Treasure Vault Can Be Opened: Large‐Scale Genome Skimming Works Well Using Herbarium and Silica Gel Dried Material.” Plants 9: 432. 10.3390/plants9040432 PMC7238428 32244605

[men70202-bib-0004] Andermann, T. , M. F. Torres Jiménez , P. Matos‐Maraví , et al. 2020. “A Guide to Carrying Out a Phylogenomic Target Sequence Capture Project.” Frontiers in Genetics 10: 1407. 10.3389/fgene.2019.01407 PMC7047930 32153629

[men70202-bib-0005] Andrews, S. 2010. “FastQC: A Quality Control Analysis Tool for High Throughput Sequencing Data.” https://www.bioinformatics.babraham.ac.uk/projects/fastqc/.

[men70202-bib-0006] Antonelli, A. , J. J. Clarkson , K. Kainulainen , et al. 2021. “Settling a Family Feud: A High‐Level Phylogenomic Framework for the Gentianales Based on 353 Nuclear Genes and Partial Plastomes.” American Journal of Botany 108: 1143–1165. 10.1002/ajb2.1697 34254285

[men70202-bib-0007] Antonelli, A. , A. Zizka , F. A. Carvalho , et al. 2018. “Amazonia Is the Primary Source of Neotropical Biodiversity.” Proceedings of the National Academy of Sciences of the United States of America 115: 6034–6039. 10.1073/pnas.1713819115 PMC6003360 29760058

[men70202-bib-0008] Baker, W. J. , P. Bailey , V. Barber , et al. 2022. “A Comprehensive Phylogenomic Platform for Exploring the Angiosperm Tree of Life.” Systematic Biology 71: 301–319. 10.1093/sysbio/syab035 PMC8830076 33983440

[men70202-bib-0009] Baker, W. J. , S. Dodsworth , F. Forest , et al. 2021. “Exploring Angiosperms353: An Open, Community Toolkit for Collaborative Phylogenomic Research on Flowering Plants.” American Journal of Botany 108: 1059–1065. 10.1002/ajb2.1703 34293179

[men70202-bib-0010] Bateman, R. M. , P. M. Hollingsworth , J. Preston , L. Yi‐Bo , A. M. Pridgeon , and M. W. Chase . 2003. “Molecular Phylogenetics and Evolution of Orchidinae and Selected Habenariinae (Orchidaceae).” Botanical Journal of the Linnean Society 142: 1–40.

[men70202-bib-0011] BDM Coordination Office . 2014. “Swiss Biodiversity Monitoring BDM. Description of Methods and Indicators.” Federal Office for the Environment, Bern. Environmental studies n° 1410.

[men70202-bib-0013] Bolger, A. M. , M. Lohse , and B. Usadel . 2014. “Trimmomatic: A Flexible Trimmer for Illumina Sequence Data.” Bioinformatics 30: 2114–2120. 10.1093/bioinformatics/btu170 PMC4103590 24695404

[men70202-bib-0014] Bornand, C. , A. Gygax , P. Juillerat , et al. 2016. Liste Rouge Plantes Vasculaires. Espèces Menacées en Suisse. Office fédéral de l'environnement, Berne et Info Flora, Genève. L'environnement pratique n° 1621.

[men70202-bib-0015] Brewer, G. E. , J. J. Clarkson , O. Maurin , et al. 2019. “Factors Affecting Targeted Sequencing of 353 Nuclear Genes From Herbarium Specimens Spanning the Diversity of Angiosperms.” Frontiers in Plant Science 10: 1102. 10.3389/fpls.2019.01102 PMC6759688 31620145

[men70202-bib-0016] Caetano Wyler, S. , and Y. Naciri . 2016. “Evolutionary Histories Determine DNA Barcoding Success in Vascular Plants: Seven Case Studies Using Intraspecific Broad Sampling of Closely Related Species.” BMC Evolutionary Biology 16: 103. 10.1186/s12862-016-0678-0 PMC4866073 27178437

[men70202-bib-0017] Cano, Á. , F. W. Stauffer , T. Andermann , et al. 2022. “Recent and Local Diversification of Central American Understorey Palms.” Global Ecology and Biogeography 31: 1513–1525.

[men70202-bib-0018] Carter, K. A. , A. Liston , N. V. Bassil , et al. 2019. “Target Capture Sequencing Unravels Rubus Evolution.” Frontiers in Plant Science 10: 1615. 10.3389/fpls.2019.01615 PMC6933950 31921259

[men70202-bib-0019] Cavender‐Bares, J. , K. H. Kozak , P. V. A. Fine , and S. W. Kembel . 2009. “The Merging of Community Ecology and Phylogenetic Biology.” Ecology Letters 12: 693–715. 10.1111/j.1461-0248.2009.01314.x 19473217

[men70202-bib-0021] Charmet, G. , C. Ravel , and F. Balfourier . 1997. “Phylogenetic Analysis in the Festuca‐Lolium Complex Using Molecular Markers and ITS rDNA.” Theoretical and Applied Genetics 94: 1038–1046.

[men70202-bib-0023] Coissac, E. , P. M. Hollingsworth , S. Lavergne , and P. Taberlet . 2016. “From Barcodes to Genomes: Extending the Concept of DNA Barcoding.” Molecular Ecology 25: 1423–1428. 10.1111/mec.13549 26821259

[men70202-bib-0024] Crowl, A. A. , E. Mavrodiev , G. Mansion , et al. 2014. “Phylogeny of Campanuloideae (Campanulaceae) With Emphasis on the Utility of Nuclear Pentatricopeptide Repeat (PPR) Genes (EJ Louis, ed.).” PLoS One 9: e94199. 10.1371/journal.pone.0094199 PMC3981779 24718519

[men70202-bib-0025] Currat, M. , M. Ruedi , R. J. Petit , and L. Excoffier . 2008. “The Hidden Side of Invasions: Massive Introgression by Local Genes.” Evolution; International Journal of Organic Evolution 62: 1908–1920. 10.1111/j.1558-5646.2008.00413.x 18452573

[men70202-bib-0026] Danecek, P. , J. K. Bonfield , J. Liddle , et al. 2021. “Twelve Years of SAMtools and BCFtools.” GigaScience 10: giab008. 10.1093/gigascience/giab008 PMC7931819 33590861

[men70202-bib-0027] De La Harpe, M. , J. Hess , O. Loiseau , N. Salamin , C. Lexer , and M. Paris . 2019. “A Dedicated Target Capture Approach Reveals Variable Genetic Markers Across Micro‐ and Macro‐Evolutionary Time Scales in Palms.” Molecular Ecology Resources 19: 221–234. 10.1111/1755-0998.12945 30240120

[men70202-bib-0028] Dexter, K. G. , M. Lavin , B. M. Torke , et al. 2017. “Dispersal Assembly of Rain Forest Tree Communities Across the Amazon Basin.” Proceedings of the National Academy of Sciences of the United States of America 114: 2645–2650. 10.1073/pnas.1613655114 PMC5347625 28213498

[men70202-bib-0029] Dodsworth, S. , L. Pokorny , M. G. Johnson , et al. 2019. “Hyb‐Seq for Flowering Plant Systematics.” Trends in Plant Science 24: 887–891. 10.1016/j.tplants.2019.07.011 31477409

[men70202-bib-0030] Doyle, J. J. , and J. L. Doyle . 1987. “A Rapid DNA Isolation Procedure for Small Quantities of Fresh Leaf Tissue.” Phytochemical Bulletin 19: 11–15.

[men70202-bib-0031] Drummond, A. J. , and A. Rambaut . 2007. “BEAST: Bayesian Evolutionary Analysis by Sampling Trees.” BMC Evolutionary Biology 7: 214. 10.1186/1471-2148-7-214 PMC2247476 17996036

[men70202-bib-0032] Ewels, P. , M. Magnusson , S. Lundin , and M. Käller . 2016. “MultiQC: Summarize Analysis Results for Multiple Tools and Samples in a Single Report.” Bioinformatics 32: 3047–3048. 10.1093/bioinformatics/btw354 PMC5039924 27312411

[men70202-bib-0033] Faircloth, B. , and T. Glenn . 2011. “Serapure—v 2.2.” https://ethanomics.files.wordpress.com/2012/08/serapure_v2‐2.pdf.

[men70202-bib-0034] Forest, F. , R. Grenyer , M. Rouget , et al. 2007. “Preserving the Evolutionary Potential of Floras in Biodiversity Hotspots.” Nature 445: 757–760. 10.1038/nature05587 17301791

[men70202-bib-0035] Forest, F. , J. Moat , E. Baloch , et al. 2018. “Gymnosperms on the EDGE.” Scientific Reports 8: 6053. 10.1038/s41598-018-24365-4 PMC5902588 29662101

[men70202-bib-0036] Franke, S. , R. Brandl , C. Heibl , et al. 2020. “Predicting Regional Hotspots of Phylogenetic Diversity Across Multiple Species Groups.” Diversity and Distributions 26: 1305–1314.

[men70202-bib-0037] Fuentes‐Bazan, S. , P. Uotila , and T. Borsch . 2012. “A Novel Phylogeny‐Based Generic Classification for *Chenopodium* Sensu Lato, and a Tribal Rearrangement of *Chenopodioideae (Chenopodiaceae)* .” Willdenowia 42: 5–24.

[men70202-bib-0038] Gaut, B. S. , L. P. Tredway , C. Kubik , R. L. Gaut , and W. Meyer . 2000. “Phylogenetic Relationships and Genetic Diversity Among Members of the *Festuca‐Lolium* Complex (Poaceae) Based on ITS Sequence Data.” Plant Systematics and Evolution 224: 33–53.

[men70202-bib-0039] Gebauer, S. , J. R. Starr , and M. H. Hoffmann . 2014. “Parallel and Convergent Diversification in Two Northern Hemispheric Species‐Rich Carex Lineages (Cyperaceae).” Organisms Diversity & Evolution 14: 247–258.

[men70202-bib-0042] Grass Phylogeny Working Group III . 2025. “A Nuclear Phylogenomic Tree of Grasses (Poaceae) Recovers Current Classification Despite Gene Tree Incongruence.” New Phytologist 245: 818–834. 10.1111/nph.20263 PMC11655423 39568153

[men70202-bib-0043] Guo, X. , D. Fang , S. K. Sahu , et al. 2021. “Chloranthus Genome Provides Insights Into the Early Diversification of Angiosperms.” Nature Communications 12: 6930. 10.1038/s41467-021-26922-4 PMC8626473 34836973

[men70202-bib-0044] Haigh, A. L. , M. Gibernau , O. Maurin , et al. 2023. “Target Sequence Data Shed New Light on the Infrafamilial Classification of Araceae.” American Journal of Botany 110: e16117. 10.1002/ajb2.16117 36480380

[men70202-bib-0045] Hale, H. , E. M. Gardner , J. Viruel , L. Pokorny , and M. G. Johnson . 2020. “Strategies for Reducing Per‐Sample Costs in Target Capture Sequencing for Phylogenomics and Population Genomics in Plants.” Applications in Plant Sciences 8: e11337. 10.1002/aps3.11337 PMC7186906 32351798

[men70202-bib-0046] Heled, J. , and A. J. Drummond . 2010. “Bayesian Inference of Species Trees From Multilocus Data.” Molecular Biology and Evolution 27: 570–580. 10.1093/molbev/msp274 PMC2822290 19906793

[men70202-bib-0047] Hinchliff, C. E. , and S. A. Smith . 2014. “Some Limitations of Public Sequence Data for Phylogenetic Inference (In Plants).” PLoS One 9: e98986. 10.1371/journal.pone.0098986 PMC4085032 24999823

[men70202-bib-0048] Honeck, E. , A. Moilanen , B. Guinaudeau , et al. 2020. “Implementing Green Infrastructure for the Spatial Planning of Peri‐Urban Areas in Geneva, Switzerland.” Sustainability 12: 1387.

[men70202-bib-0049] Inda, L. A. , M. Pimentel , and M. W. Chase . 2012. “Phylogenetics of Tribe Orchideae (Orchidaceae: Orchidoideae) Based on Combined DNA Matrices: Inferences Regarding Timing of Diversification and Evolution of Pollination Syndromes.” Annals of Botany 110: 71–90. 10.1093/aob/mcs083 PMC3380586 22539542

[men70202-bib-0050] Jantzen, J. R. , W. M. Whitten , K. M. Neubig , L. C. Majure , D. E. Soltis , and P. S. Soltis . 2019. “Effects of Taxon Sampling and Tree Reconstruction Methods on Phylodiversity Metrics.” Ecology and Evolution 9: 9479–9499. 10.1002/ece3.5425 PMC6745870 31534670

[men70202-bib-0051] Jin, Y. , and H. Qian . 2022. “V.PhyloMaker2: An Updated and Enlarged R Package That Can Generate Very Large Phylogenies for Vascular Plants.” Plant Diversity 44: 335–339. 10.1016/j.pld.2022.05.005 PMC9363651 35967255

[men70202-bib-0052] Johnson, M. G. , L. Pokorny , S. Dodsworth , et al. 2019. “A Universal Probe Set for Targeted Sequencing of 353 Nuclear Genes From Any Flowering Plant Designed Using k‐Medoids Clustering.” Systematic Biology 68: 594–606. 10.1093/sysbio/syy086 PMC6568016 30535394

[men70202-bib-0053] Juillerat, P. , B. Bäumler , C. Bornand , et al. 2017. “Checklist 2017 der Gefässpflanzenflora der Schweiz/de la Flore Vasculaire de la Suisse/Della Flora Vascolare Della Svizzera.” Info Flora, Centre de données et d'infromations sur la flore de Suisse.

[men70202-bib-0054] Karl, R. , and M. A. Koch . 2013. “A World‐Wide Perspective on Crucifer Speciation and Evolution: Phylogenetics, Biogeography and Trait Evolution in Tribe Arabideae.” Annals of Botany 112: 983–1001. 10.1093/aob/mct165 PMC3783230 23904444

[men70202-bib-0055] Kim, S.‐T. , and M. J. Donoghue . 2008. “Molecular Phylogeny of *Persicaria* (Persicarieae, Polygonaceae).” Systematic Botany 33: 77–86.

[men70202-bib-0056] Knapp, S. , I. Kühn , O. Schweiger , and S. Klotz . 2008. “Challenging Urban Species Diversity: Contrasting Phylogenetic Patterns Across Plant Functional Groups in Germany.” Ecology Letters 11: 1054–1064. 10.1111/j.1461-0248.2008.01217.x 18616547

[men70202-bib-0058] Lavergne, S. , C. Pouchon , C. Roquet , et al. 2025. “Towards a Comprehensive Barcoding and Phylogenomic Reference for the European Arctic‐Alpine Flora.” Botany Letters 172: 495–507.

[men70202-bib-0059] Lee, A. K. , I. S. Gilman , M. Srivastav , A. D. Lerner , M. J. Donoghue , and W. L. Clement . 2021. “Reconstructing Dipsacales Phylogeny Using Angiosperms353: Issues and Insights.” American Journal of Botany 108: 1122–1142. 10.1002/ajb2.1695 PMC8362060 34254290

[men70202-bib-0060] Letunic, I. , and P. Bork . 2021. “Interactive Tree of Life (iTOL) v5: An Online Tool for Phylogenetic Tree Display and Annotation.” Nucleic Acids Research 49: W293–W296. 10.1093/nar/gkab301 PMC8265157 33885785

[men70202-bib-0061] Li, D. , L. Trotta , H. E. Marx , et al. 2019. “For Common Community Phylogenetic Analyses, Go Ahead and Use Synthesis Phylogenies.” Ecology 100: e02788. 10.1002/ecy.2788 PMC7079099 31225900

[men70202-bib-0062] Li, H. , and R. Durbin . 2009. “Fast and Accurate Short Read Alignment With Burrows–Wheeler Transform.” Bioinformatics 25: 1754–1760. 10.1093/bioinformatics/btp324 PMC2705234 19451168

[men70202-bib-0150] Liu, B. B. , Z. Y. Ma , C. Ren , et al. 2021. “Capturing Single‐Copy Nuclear Genes, Organellar Genomes, and Nuclear Ribosomal DNA from Deep Genome Skimming Data for Plant Phylogenetics: A Case Study in Vitaceae.” Journal of Systematics and Evolution 59: 1124–1138.

[men70202-bib-0063] Liu, B.‐B. , C. S. Campbell , D.‐Y. Hong , and J. Wen . 2020. “Phylogenetic Relationships and Chloroplast Capture in the Amelanchier‐Malacomeles‐Peraphyllum Clade (Maleae, Rosaceae): Evidence From Chloroplast Genome and Nuclear Ribosomal DNA Data Using Genome Skimming.” Molecular Phylogenetics and Evolution 147: 106784. 10.1016/j.ympev.2020.106784 32135308

[men70202-bib-0064] Lo, E. Y. Y. , and M. J. Donoghue . 2012. “Expanded Phylogenetic and Dating Analyses of the Apples and Their Relatives (Pyreae, Rosaceae).” Molecular Phylogenetics and Evolution 63: 230–243. 10.1016/j.ympev.2011.10.005 22293154

[men70202-bib-0065] Magallón, S. , S. Gómez‐Acevedo , L. L. Sánchez‐Reyes , and T. Hernández‐Hernández . 2015. “A Metacalibrated Time‐Tree Documents the Early Rise of Flowering Plant Phylogenetic Diversity.” New Phytologist 207: 437–453. 10.1111/nph.13264 25615647

[men70202-bib-0066] Mai, U. , and S. Mirarab . 2018. “TreeShrink: Fast and Accurate Detection of Outlier Long Branches in Collections of Phylogenetic Trees.” BMC Genomics 19: 272. 10.1186/s12864-018-4620-2 PMC5998883 29745847

[men70202-bib-0067] Mansion, G. , G. Parolly , A. A. Crowl , et al. 2012. “How to Handle Speciose Clades? Mass Taxon‐Sampling as a Strategy Towards Illuminating the Natural History of Campanula (Campanuloideae).” PLoS One 7: e50076. 10.1371/journal.pone.0050076 PMC3509159 23209646

[men70202-bib-0068] Martín‐Bravo, S. , P. Jiménez‐Mejías , T. Villaverde , et al. 2019. “A Tale of Worldwide Success: Behind the Scenes of *Carex* (Cyperaceae) Biogeography and Diversification.” Journal of Systematics and Evolution 57: 695–718.

[men70202-bib-0069] Maurin, O. , A. Anest , S. Bellot , et al. 2021. “A Nuclear Phylogenomic Study of the Angiosperm Order Myrtales, Exploring the Potential and Limitations of the Universal Angiosperms353 Probe Set.” American Journal of Botany 108: 1087–1111. 10.1002/ajb2.1699 34297852

[men70202-bib-0070] McDonnell, A. J. , W. J. Baker , S. Dodsworth , et al. 2021. “Exploring Angiosperms353: Developing and Applying a Universal Toolkit for Flowering Plant Phylogenomics.” Applications in Plant Sciences 9: aps3.11443. 10.1002/aps3.11443 PMC8312743 34336400

[men70202-bib-0071] McLay, T. G. B. , J. L. Birch , B. F. Gunn , et al. 2021. “New Targets Acquired: Improving Locus Recovery From the Angiosperms353 Probe Set.” Applications in Plant Sciences 9: aps3.11420. 10.1002/aps3.11420 PMC8312740 34336399

[men70202-bib-0072] Meng, K.‐K. , S.‐F. Chen , K.‐W. Xu , et al. 2021. “Phylogenomic Analyses Based on Genome‐Skimming Data Reveal Cyto‐Nuclear Discordance in the Evolutionary History of Cotoneaster (Rosaceae).” Molecular Phylogenetics and Evolution 158: 107083. 10.1016/j.ympev.2021.107083 33516804

[men70202-bib-0073] Merckx, V. S. F. T. , K. P. Hendriks , K. K. Beentjes , et al. 2015. “Evolution of Endemism on a Young Tropical Mountain.” Nature 524: 347–350. 10.1038/nature14949 26266979

[men70202-bib-0074] Minh, B. Q. , H. A. Schmidt , O. Chernomor , et al. 2020. “IQ‐TREE 2: New Models and Efficient Methods for Phylogenetic Inference in the Genomic Era.” Molecular Biology and Evolution 37: 1530–1534. 10.1093/molbev/msaa015 PMC7182206 32011700

[men70202-bib-0075] Mishler, B. D. , N. Knerr , C. E. González‐Orozco , A. H. Thornhill , S. W. Laffan , and J. T. Miller . 2014. “Phylogenetic Measures of Biodiversity and Neo‐ and Paleo‐Endemism in Australian Acacia.” Nature Communications 5: 4473. 10.1038/ncomms5473 25034856

[men70202-bib-0076] Mo, Z.‐Q. , C.‐N. Fu , A. D. Twyford , et al. 2025. “Evaluating the Utility of Deep Genome Skimming for Phylogenomic Analyses: A Case Study in the Species‐Rich Genus Rhododendron.” Plant Diversity 47: 593–603. 10.1016/j.pld.2025.04.006 PMC12302500 40734829

[men70202-bib-0077] Molina‐Venegas, R. , I. Ramos‐Gutiérrez , and J. C. Moreno‐Saiz . 2020. “Phylogenetic Patterns of Extinction Risk in the Endemic Flora of a Mediterranean Hotspot as a Guiding Tool for Preemptive Conservation Actions.” Frontiers in Ecology and Evolution 8: 571587.

[men70202-bib-0078] Mombrial, F. , M. Chevalier , E. Favre , et al. 2020. “Liste Rouge des Plantes Vasculaires du Canton de Genève.” CJB‐OCAN.

[men70202-bib-0081] Naciri, Y. , S. Caetano , and N. Salamin . 2012. “Plant DNA Barcodes and the Influence of Gene Flow.” Molecular Ecology Resources 12: 575–580. 10.1111/j.1755-0998.2012.03130.x 22394382

[men70202-bib-0082] Naciri, Y. , and H. P. Linder . 2015. “Species Delimitation and Relationships: The Dance of the Seven Veils.” Taxon 64: 3–16.

[men70202-bib-0083] Naciri, Y. , and H. P. Linder . 2020. “The Genetics of Evolutionary Radiations.” Biological Reviews 95: 1055–1072. 10.1111/brv.12598 32233014

[men70202-bib-0084] Neuwirth, E. 2022. “RColorBrewer: ColorBrewer Palettes.”

[men70202-bib-0149] OFEV . 2022. Les régions biogéographiques de la Suisse. 1re édition actualisée 2022. 1re parution 2001. Office fédéral de l’environnement, Berne. Connaissance de l’environnement n° 2214.

[men70202-bib-0085] One Thousand Plant Transcriptomes Initiative . 2019. “One Thousand Plant Transcriptomes and the Phylogenomics of Green Plants.” Nature 574: 679–685. 10.1038/s41586-019-1693-2 PMC6872490 31645766

[men70202-bib-0089] Pérez‐Escobar, O. A. , A. Zizka , M. A. Bermúdez , et al. 2022. “The Andes Through Time: Evolution and Distribution of Andean Floras.” Trends in Plant Science 27: 364–378. 10.1016/j.tplants.2021.09.010 35000859

[men70202-bib-0090] Pipins, S. , J. E. M. Baillie , A. Bowmer , L. J. Pollock , N. Owen , and R. Gumbs . 2024. “Advancing EDGE Zones to Identify Spatial Conservation Priorities of Tetrapod Evolutionary History.” Nature Communications 15: 7672. 10.1038/s41467-024-51992-5 PMC11377708 39237497

[men70202-bib-0092] Pouchon, C. , and C. G. Boluda . 2023. “REFMAKER: Make Your Own Reference to Target Nuclear Loci in Low Coverage Genome Skimming Libraries. Phylogenomic Application in Sapotaceae.” Molecular Phylogenetics and Evolution 186: 107826. 10.1016/j.ympev.2023.107826 37257798

[men70202-bib-0093] Pouchon, C. , F. Boyer , C. Roquet , et al. 2022. “ORTHOSKIM: In Silico Sequence Capture From Genomic and Transcriptomic Libraries for Phylogenomic and Barcoding Applications.” Molecular Ecology Resources 22: 2018–2037. 10.1111/1755-0998.13584 35015377

[men70202-bib-0094] Qian, H. , and Y. Jin . 2016. “An Updated Megaphylogeny of Plants, a Tool for Generating Plant Phylogenies and an Analysis of Phylogenetic Community Structure.” Journal of Plant Ecology 9: 233–239.

[men70202-bib-0095] Qian, H. , and Y. Jin . 2021. “Are Phylogenies Resolved at the Genus Level Appropriate for Studies on Phylogenetic Structure of Species Assemblages?” Plant Diversity 43: 255–263. 10.1016/j.pld.2020.11.005 PMC8390917 34485767

[men70202-bib-0096] Qian, H. , R. E. Ricklefs , and W. Thuiller . 2021. “Evolutionary Assembly of Flowering Plants Into Sky Islands.” Nature Ecology & Evolution 5: 640–646. 10.1038/s41559-021-01423-1 33782577

[men70202-bib-0097] Ramírez‐Barahona, S. , H. Sauquet , and S. Magallón . 2020. “The Delayed and Geographically Heterogeneous Diversification of Flowering Plant Families.” Nature Ecology & Evolution 4: 1232–1238. 10.1038/s41559-020-1241-3 32632260

[men70202-bib-0098] Ratnasingham, S. , and P. D. N. Hebert . 2007. “BOLD: The Barcode of Life Data System.” Molecular Ecology Notes 7: 355–364. https://www.barcodinglife.org. 10.1111/j.1471-8286.2007.01678.x PMC1890991 18784790

[men70202-bib-0099] Revell, L. J. 2012. “Phytools: An R Package for Phylogenetic Comparative Biology (and Other Things).” Methods in Ecology and Evolution 3: 217–223.

[men70202-bib-0100] Rieseberg, L. H. , and D. E. Soltis . 1991. “Phylogenetic Consequences of Cytoplasmic Gene Flow in Plants.” Evolutionary Trends in Plants 5: 65–84.

[men70202-bib-0101] Rohland, N. , and D. Reich . 2012. “Cost‐Effective, High‐Throughput DNA Sequencing Libraries for Multiplexed Target Capture.” Genome Research 22: 939–946. 10.1101/gr.128124.111 PMC3337438 22267522

[men70202-bib-0102] Saladin, B. , L. Pellissier , C. H. Graham , M. P. Nobis , N. Salamin , and N. E. Zimmermann . 2020. “Rapid Climate Change Results in Long‐Lasting Spatial Homogenization of Phylogenetic Diversity.” Nature Communications 11: 4663. 10.1038/s41467-020-18343-6 PMC7495423 32938914

[men70202-bib-0104] Sauquet, H. , S. Y. W. Ho , M. A. Gandolfo , et al. 2012. “Testing the Impact of Calibration on Molecular Divergence Times Using a Fossil‐Rich Group: The Case of Nothofagus (Fagales).” Systematic Biology 61: 289–313. 10.1093/sysbio/syr116 22201158

[men70202-bib-0105] Sauquet, H. , S. Ramírez‐Barahona , and S. Magallón . 2022. “What Is the Age of Flowering Plants?” Journal of Experimental Botany 73: 3840–3853. 10.1093/jxb/erac130 35438718

[men70202-bib-0106] Sauquet, H. , P. H. Weston , C. L. Anderson , et al. 2009. “Contrasted Patterns of Hyperdiversification in Mediterranean Hotspots.” Proceedings of the National Academy of Sciences of the United States of America 106: 221–225. 10.1073/pnas.0805607106 PMC2629191 19116275

[men70202-bib-0107] Schaefer, H. , P. Hechenleitner , A. Santos‐Guerra , et al. 2012. “Systematics, Biogeography, and Character Evolution of the Legume Tribe Fabeae With Special Focus on the Middle‐Atlantic Island Lineages.” BMC Evolutionary Biology 12: 250. 10.1186/1471-2148-12-250 PMC3547781 23267563

[men70202-bib-0108] Simão, F. A. , R. M. Waterhouse , P. Ioannidis , E. V. Kriventseva , and E. M. Zdobnov . 2015. “BUSCO: Assessing Genome Assembly and Annotation Completeness With Single‐Copy Orthologs.” Bioinformatics 31: 3210–3212. 10.1093/bioinformatics/btv351 26059717

[men70202-bib-0109] Simon, M. F. , R. Grether , L. P. De Queiroz , C. Skema , R. T. Pennington , and C. E. Hughes . 2009. “Recent Assembly of the Cerrado, a Neotropical Plant Diversity Hotspot, by In Situ Evolution of Adaptations to Fire.” Proceedings of the National Academy of Sciences of the United States of America 106: 20359–20364. 10.1073/pnas.0903410106 PMC2787167 19918050

[men70202-bib-0110] Simpson, L. , D. Cantrill , M. Byrne , et al. 2024. “The Genomics for Australian Plants (GAP) Framework Initiative—Developing Genomic Resources for Understanding the Evolution and Conservation of the Australian Flora.” Australian Systematic Botany 38: SB24022.

[men70202-bib-0111] Slimp, M. , L. D. Williams , H. Hale , and M. G. Johnson . 2021. “On the Potential of Angiosperms353 for Population Genomic Studies.” Applications in Plant Sciences 9: aps3.11419. 10.1002/aps3.11419 PMC8312745 34336401

[men70202-bib-0112] Small, R. L. , R. C. Cronn , and J. F. Wendel . 2004. “Use of Nuclear Genes for Phylogeny Reconstruction in Plants.” Australian Systematic Botany 17: 145–170.

[men70202-bib-0114] Smith, S. A. , and J. W. Brown . 2018. “Constructing a Broadly Inclusive Seed Plant Phylogeny.” American Journal of Botany 105: 302–314. 10.1002/ajb2.1019 29746720

[men70202-bib-0115] Smith, S. A. , and B. C. O'Meara . 2012. “treePL: Divergence Time Estimation Using Penalized Likelihood for Large Phylogenies.” Bioinformatics 28: 2689–2690. 10.1093/bioinformatics/bts492 22908216

[men70202-bib-0116] Smyčka, J. , C. Roquet , M. Boleda , et al. 2022. “Tempo and Drivers of Plant Diversification in the European Mountain System.” Nature Communications 13: 2750. 10.1038/s41467-022-30394-5 PMC9117672 35585056

[men70202-bib-0117] Sochor, M. , R. J. Vašut , T. F. Sharbel , and B. Trávníček . 2015. “How Just a Few Makes a Lot: Speciation via Reticulation and Apomixis on Example of European Brambles (Rubus Subgen. Rubus, Rosaceae).” Molecular Phylogenetics and Evolution 89: 13–27. 10.1016/j.ympev.2015.04.007 25882835

[men70202-bib-0118] Soltis, D. E. , and R. K. Kuzoff . 1995. “Discordance Between Nuclear and Chloroplast Phylogenies in the *Heuchera* Group (Saxifragaceae).” Evolution 49: 727–742. 10.1111/j.1558-5646.1995.tb02309.x 28565145

[men70202-bib-0119] Spalink, D. , R. Kriebel , P. Li , et al. 2018. “Spatial Phylogenetics Reveals Evolutionary Constraints on the Assembly of a Large Regional Flora.” American Journal of Botany 105: 1938–1950. 10.1002/ajb2.1191 30408151

[men70202-bib-0120] Stebbins, G. L. 1985. “Polyploidy, Hybridization, and the Invasion of New Habitats.” Annals of the Missouri Botanical Garden 72: 824.

[men70202-bib-0121] Stefani, S. , L. Champoud , L. Fazan , et al. 2025. “Genetic Divergence of *Clematis alpina* in the Swiss Prealps: A Tale of the Margins.” Alpine Botany 135: 167–185.

[men70202-bib-0122] Stull, G. W. , P. S. Soltis , D. E. Soltis , M. A. Gitzendanner , and S. A. Smith . 2020. “Nuclear Phylogenomic Analyses of Asterids Conflict With Plastome Trees and Support Novel Relationships Among Major Lineages.” American Journal of Botany 107: 790–805. 10.1002/ajb2.1468 32406108

[men70202-bib-0124] The Global Carex Group , P. Jiménez‐Mejías , M. Hahn , et al. 2016. “Megaphylogenetic Specimen‐Level Approaches to the *Carex* (Cyperaceae) Phylogeny Using ITS, ETS, and *matK* Sequences: Implications for Classification.” Systematic Botany 41: 500–518.

[men70202-bib-0125] Thomas, S. K. , X. Liu , Z. Du , et al. 2021. “Comprehending Cornales: Phylogenetic Reconstruction of the Order Using the Angiosperms353 Probe Set.” American Journal of Botany 108: 1112–1121. 10.1002/ajb2.1696 PMC8361741 34263456

[men70202-bib-0127] Thornhill, A. H. , B. G. Baldwin , W. A. Freyman , et al. 2017. “Spatial Phylogenetics of the Native California Flora.” BMC Biology 15: 96. 10.1186/s12915-017-0435-x PMC5658987 29073895

[men70202-bib-0128] Thornhill, A. H. , B. D. Mishler , N. J. Knerr , et al. 2016. “Continental‐Scale Spatial Phylogenetics of Australian Angiosperms Provides Insights Into Ecology, Evolution and Conservation.” Journal of Biogeography 43: 2085–2098.

[men70202-bib-0129] Thuiller, W. , S. Lavergne , C. Roquet , I. Boulangeat , B. Lafourcade , and M. B. Araujo . 2011. “Consequences of Climate Change on the Tree of Life in Europe.” Nature 470: 531–534. 10.1038/nature09705 21326204

[men70202-bib-0130] Torrecilla, P. , and P. Catalán . 2002. “Phylogeny of Broad‐Leaved and Fine‐Leaved Festuca Lineages (Poaceae) Based on Nuclear ITS Sequences.” Systematic Botany 27: 241–251.

[men70202-bib-0131] Tsitrone, A. , M. Kirkpatrick , and D. A. Levin . 2003. “A Model for Chloroplast Capture.” Evolution 57: 1776–1782. 10.1111/j.0014-3820.2003.tb00585.x 14503619

[men70202-bib-0132] Vilanova, S. , D. Alonso , P. Gramazio , et al. 2020. “SILEX: A Fast and Inexpensive High‐Quality DNA Extraction Method Suitable for Multiple Sequencing Platforms and Recalcitrant Plant Species.” Plant Methods 16: 110. 10.1186/s13007-020-00652-y PMC7419208 32793297

[men70202-bib-0133] Walker, J. F. , Y. Yang , T. Feng , et al. 2018. “From Cacti to Carnivores: Improved Phylotranscriptomic Sampling and Hierarchical Homology Inference Provide Further Insight Into the Evolution of Caryophyllales.” American Journal of Botany 105: 446–462. 10.1002/ajb2.1069 29738076

[men70202-bib-0134] Warnes, G. R. , B. Bolker , L. Bonebakker , et al. 2009. gplots: Various R Programming Tools for Plotting Data. CRAN.

[men70202-bib-0135] Webb, C. O. 2000. “Exploring the Phylogenetic Structure of Ecological Communities: An Example for Rain Forest Trees.” American Naturalist 156: 145–155. 10.1086/303378 10856198

[men70202-bib-0136] Weber, C. 1966. “Catalogue Dynamique de la Flore de Genève.” Boissiera 12: 1–259.

[men70202-bib-0137] Winter, M. , O. Schweiger , S. Klotz , et al. 2009. “Plant Extinctions and Introductions Lead to Phylogenetic and Taxonomic Homogenization of the European Flora.” Proceedings of the National Academy of Sciences of the United States of America 106: 21721–21725. 10.1073/pnas.0907088106 PMC2792159 20007367

[men70202-bib-0138] Wolfe, K. H. , C. W. Morden , and J. D. Palmer . 1992. “Function and Evolution of a Minimal Plastid Genome From a Nonphotosynthetic Parasitic Plant.” Proceedings of the National Academy of Sciences of the United States of America 89: 10648–10652. 10.1073/pnas.89.22.10648 PMC50398 1332054

[men70202-bib-0139] Wootton, L. M. , F. C. Boucher , C. Pouchon , et al. 2025. “The Late Rise of Sky‐Island Vegetation in the European Alps.” Nature Plants 11: 1142–1153. 10.1038/s41477-025-02001-0. 40457047

[men70202-bib-0140] Xing, Y. , and R. H. Ree . 2017. “Uplift‐Driven Diversification in the Hengduan Mountains, a Temperate Biodiversity Hotspot.” Proceedings of the National Academy of Sciences of the United States of America 114: E3444–E3451. 10.1073/pnas.1616063114 PMC5410793 28373546

[men70202-bib-0141] Xu, C. , and D. Hong . 2021. “Phylogenetic Analyses Confirm Polyphyly of the Genus *Campanula* (Campanulaceae s. Str.), Leading to a Proposal for Generic Reappraisal.” Journal of Systematics and Evolution 59: 475–489.

[men70202-bib-0142] Yang, L. , D. Su , X. Chang , et al. 2020. “Phylogenomic Insights Into Deep Phylogeny of Angiosperms Based on Broad Nuclear Gene Sampling.” Plant Communications 1: 100027. 10.1016/j.xplc.2020.100027 PMC7747974 33367231

[men70202-bib-0143] Zanne, A. E. , D. C. Tank , W. K. Cornwell , et al. 2014. “Three Keys to the Radiation of Angiosperms Into Freezing Environments.” Nature 506: 89–92. 10.1038/nature12872 24362564

[men70202-bib-0144] Zeng, L. , Q. Zhang , R. Sun , H. Kong , N. Zhang , and H. Ma . 2014. “Resolution of Deep Angiosperm Phylogeny Using Conserved Nuclear Genes and Estimates of Early Divergence Times.” Nature Communications 5: 4956. 10.1038/ncomms5956 PMC4200517 25249442

[men70202-bib-0145] Zhang, C. , and S. Mirarab . 2022. “Weighting by Gene Tree Uncertainty Improves Accuracy of Quartet‐Based Species Trees.” Molecular Biology and Evolution 39: 215. 10.1093/molbev/msac215 PMC9750496 36201617

[men70202-bib-0146] Zhang, G. , J. Yang , C. Zhang , et al. 2024. “Nuclear Phylogenomics of Asteraceae With Increased Sampling Provides New Insights Into Convergent Morphological and Molecular Evolution.” Plant Communications 5: 100851. 10.1016/j.xplc.2024.100851 PMC11211554 38409784

[men70202-bib-0148] Zuntini, A. R. , T. Carruthers , O. Maurin , et al. 2024. “Phylogenomics and the Rise of the Angiosperms.” Nature 629: 843–850. 10.1038/s41586-024-07324-0 PMC11111409 38658746

