# Supplementary material for: Simultaneous Inference of Flora‐Wide Nuclear and Plastid Phylogenies Using Angiosperms353 Sequence Capture Data
Source: Mol Ecol Resour. 2026 Sep 28;26(7):e70202. doi: 10.1111/1755-0998.70202 (PMC13618893; doi:10.1111/1755-0998.70202)
Supplement: Supplementary file 1 [file MEN-26-e70202-s003.docx]

**Figure S1:** Violin plots comparing the ratio of reads mapped on target compared to the total number of reads for all samples according to several factors: A. Taxonomy at the ordinal level for orders comprising over 30 species (samples of this study only). B. Material preservation type. C. Source of raw genetic data used to recover target genes. D. Hybridization temperature used in the target enrichment step (samples of this study only). Numbers in bold on top of the violin plots indicate the average for each category. Statistical significance is indicated on the top right corner of each plot (nominal alpha = 0.1 (.), 0.05 (*), 0.01 (**), 0.001 (***), ns = not significant)

**Figure S2:** Heatmap of gene recovery efficiency, in which rows represent all 1628 samples and columns represent the 353 target genes hierarchically clustered into three groups with different gene recovery efficiencies. A darker colour indicates that a higher percentage of the target gene has been recovered. On the left side of the heatmap, the cladogram represents the phylogenetic position of samples included in the heatmap. The coloured bar drawn next to the cladogram indicates boundaries between orders present in the phylogeny (see Figure 3 for more details). On the right side of the heatmap, barplots indicate the number of genes recovered per sample, with silicagel preserved samples in green and herbarium preserved samples in orange.

**Figure S3:** Heatmap of gene recovery efficiency, in which rows represent all 1628 samples, ordered according to their phylogenetic position, and columns represent the 79 plastid genes.

**Figure S4:** Weighted-ASTRAL multi-species coalescent phylogeny of the Geneva flora from 349 nuclear gene trees with the hybrid-weighting mode. The coloured circle around the phylogeny delimits all 41 orders of angiosperms plus the gymnosperms outgroup. The coloured circle drawn around the tree indicates boundaries between orders present in the phylogeny. The outer barplots represent the number of plastid loci retrieved for each sample. Coloured branches represent the local posterior probability (LPP) of internal nodes, red meaning low LPP and black high LPP. The Newick tree file is available in Supplementary file 2.

**Figure S5:** IQ-TREE Maximum likelihood phylogeny for angiosperms of the Geneva flora based on the concatenation of 77 plastid genes for 1532 taxa. The coloured circle around the phylogeny delimits all 41 orders of angiosperms plus the gymnosperms outgroup. The coloured circle drawn around the tree indicates boundaries between orders present in the phylogeny. The outer barplots represent the number of plastid loci retrieved for each sample. This phylogeny was inferred using a concatenated alignment of 56’342 positions, and the statistical support of nodes was inferred with 1’000 Ultrafast Bootstrap (UFBoot) replicates. Coloured branches indicate the support of internal nodes, red meaning low support and black high support. The Newick tree file is available in Supplementary file 2.

**Figure S6**: Cophylogenetic plots of major clades between this study’s ML (left) and ASTRAL(right) nuclear phylogenies. A, Order level comparison. B, Family level comparison. Families comprising more than 30 samples are highlighted in red and detailed in Fig. S7.

**Figure S7:** Species-level cophylogenetic plots of major families (>30 samples) between the ML (left) and ASTRAL (right) nuclear phylogenies. A, Apiaceae. B, Asteraceae. C, Brassicaceae. D, Caryophyllaceae. E, Cyperaceae. F, Fabaceae. G, Lamiaceae. H, Orchidaceae. I, Plantaginaceae. J, Poaceae. K, Ranunculaceae. L, Rosaceae. Nodes with low UFBoot support (<70) or LPP (<0.7) are resolved as polytomies.

**Figure S8:** Cophylogenetic plots of major clades between this study’s ML nuclear (left) and PAFTOL young (right) phylogenies. A, Ordinal level comparison. B, Family level comparison.

**Figure S9:** Cophylogenetic plots of major clades between this study’s ML nuclear (left) and plastid (right) phylogenies. A, Ordinal level comparison. B, Family level comparison. Families comprising more than 30 samples are highlighted in red and detailed in Fig. S10.

**Figure S10:** Species-level cophylogenetic plots of major families (>30 samples) between the nuclear (left) and plastid (right) maximum likelihood phylogenies. A, Apiaceae. B, Asteraceae. C, Brassicaceae. D, Caryophyllaceae. E, Cyperaceae. F, Fabaceae. G, Lamiaceae. H, Orchidaceae. I, Plantaginaceae. J, Poaceae. K, Ranunculaceae. L, Rosaceae. Nodes with low UFBoot support (<70) are resolved as polytomies.

**Figure S11:** Plastid chronogram of the Geneva flora containing 1235 taxa. A maximum constraint of 154 Myr was used at the root node and corresponds to the estimated upper boundary of the angiosperms crown age. Red dots at nodes indicate the placement of fossil calibrations extracted from the AngioCal v.1.1 dataset. The coloured circle drawn around the tree indicates boundaries between orders present in the phylogeny. The newick tree file is available in Supplementary file 2.

**Figure S12:** Barplots of the distribution of branch lengths in the nuclear (orange) and the plastid (green) trees for families that comprise >30 samples.

**Figure S13:** GBOTB chronogram of the Geneva flora generated by pruning the GBOTB.extended.TPL backbone available in PhyloMaker. Time scale represents the estimated age of each taxa in the chronogram.

**Figure S14:** Violin plots of the difference in estimated ages of taxonomic units between the nuclear chronogram and the plastid chronogram (top); and between the nuclear chronogram and the GBOTB chronogram (bottom).

**Figure S15**: Cophylogenetic plots of the ASTRAL species trees of 136 Poaceae accessions inferred from (left) the dataset containing only the dominant OrthoFinder orthogroup for each Angiosperms353 locus, and (right) from all Angiosperms353 sequences retained by our workflow. Differences of topology are highlighted in red.

**Table S1:** Effects of factors and variables on capture efficiency. For factor effects, p-value of the appropriate statistical test is indicated, for correlations with variables, the Spearman rho value is indicated in the first row, and the p-value for the statistical significance of the correlation is indicated in the second row. Symbols in parenthesis indicate the statistical significance at the nominal alpha = 0.1 (.), 0.05 (*), 0.01 (**), 0.001 (***) levels, (ns) being not significant (two-tailed tests). Detailed results for each sample are available in Table S3.

|  | **Taxonomy (ordinal-level)** | **Material preservation type** | **Data Source** | **Hybridization temperature** | **Genomic DNA concentration** | **Library fragment size** | **Final library concentration** | **Number of reads per sample** |
| --- | --- | --- | --- | --- | --- | --- | --- | --- |
| *Number of recovered genes* | <2.2e-16 (***) | <2.2e-16 (***) | 0.4335 (NS) | 3.16e-7 (***) | ρ = -0.13 1.8e-6 (***) | ρ = 0.09 1.17e-3 (**) | ρ = -0.02 0.539 (NS) | ρ = 0.48 2.2e-16 (***) |
| *Total length of captured sequence* | <2.2e-16 (***) | <2.2e-16 (***) | 0.3248 (NS) | 5.37e-5 (***) | ρ = -0.19 4.32e-12 (***) | ρ = -0.17 9.98e-11 (***) | ρ = 0.04 0.106 (NS) | ρ = 0.45 2.2e-16 (***) |
| *Reads on target ratio* | <2.2e-16 (***) | 0.1745 (NS) | <2.2e-16 (***) | 0.0931 (.) | ρ = -0.09 9.6e-4 (***) | ρ = -0.13 2.64e-6 (***) | ρ = -0.13 1.43e-6 (***) | ρ = 0.08 1.06e-3 (**) |

**Table S4:** List of plastid genes recovered with ORTHOSKIM that were used in the phylogenetic inference.

| *accD* | *ndhB* | *petG* | *psbF* | *rpl*2 | *rps*14 |
| --- | --- | --- | --- | --- | --- |
| *atpA* | *ndhC* | *petL* | *psbH* | *rpl*20 | *rps*15 |
| *atpB* | *ndhD* | *petN* | *psbI* | *rpl*22 | *rps*16 |
| *atpE* | *ndhE* | *psaA* | *psbJ* | *rpl*23 | *rps*18 |
| *atpF* | *ndhF* | *psaB* | *psbK* | *rpl*32 | *rps*19 |
| *atpH* | *ndhG* | *psaC* | *psbL* | *rpl*33 | *rps*2 |
| *atpI* | *ndhH* | *psaI* | *psbM* | *rpl*36 | *rps*3 |
| *ccsA* | *ndhI* | *psaJ* | *psbN* | *rpoA* | *rps*4 |
| *cemA* | *ndhJ* | *psbA* | *psbT* | *rpoB* | *rps*7 |
| *clpP* | *ndhK* | *psbB* | *psbZ* | *rpoC*1 | *rps*8 |
| *infA* | *petA* | *psbC* | *rbcL* | *rpoC*2 | *ycf*3 |
| *matK* | *petB* | *psbD* | *rpl*14 | *rps*11 | *ycf*4 |
| *ndhA* | *petD* | *psbE* | *rpl*16 | *rps*12 |  |
